# In Silico Discovery of ABZI Nitrogen Heterocycle STING Agonists via 3D-QSAR, Molecular Dynamics, and AI-Based Synthesis Prediction

**DOI:** 10.3390/ph19030387

**Published:** 2026-02-28

**Authors:** Houcheng Ren, Yuhong Jin, Baipu Zhao, Xiangbing Peng, Shan Zhao, Meiting Wang

**Affiliations:** 1 The First Clinical School, Zhengzhou University, Zhengzhou 450001, China; 2 The First Affiliated Hospital, Xinxiang Medical University, Xinxiang 453003, China; 3 Henan International Joint Laboratory of Neural Information Analysis and Drug Intelligent Design, School of Medical Engineering, Xinxiang Medical University, Xinxiang 453003, China; 4 School of Basic Medical Sciences, Xinxiang Medical University, Xinxiang 453003, China; 5 Department of Theoretical Chemistry, Chemical Centre, Lund University, SE-221 00 Lund, Sweden

**Keywords:** STING agonists, ABZI, 3D-QSAR, molecular dynamics, free energy, AI synthesis prediction

## Abstract

**Background/Objectives**: The stimulator of interferon genes (STING) pathway plays a central role in innate immune signaling and represents an attractive therapeutic target for cancer immunotherapy. Amidobenzimidazole (ABZI) derivatives have emerged as promising non-nucleotide STING agonists with improved drug-like properties compared to cyclic dinucleotides. However, current ABZI compounds still exhibit limited oral bioavailability and cross-species potency discrepancies. In addition, potential systemic toxicity remains a concern, indicating the need for further structural optimization. **Methods:** In this study, a comprehensive computer-aided drug design strategy was employed to systematically investigate ABZI derivatives and identify novel STING agonists with enhanced activity and favorable pharmacokinetic profiles. A 3D quantitative structure–activity relationship (3D-QSAR) model was constructed using the Topomer CoMFA approach based on a dataset of 109 reported ABZI compounds. Guided by the contour map analysis, new chemical groups were introduced through a fragment growth method, generating a large virtual library. The library was subsequently filtered via molecular docking, molecular dynamics simulations, and MM-PBSA binding free energy calculations. **Results**: Among the newly designed ABZI compounds, five compounds displayed lower binding free energies than D59, with M13 and M44 showing reductions exceeding 6.7 kcal/mol. This work demonstrates the effectiveness of an integrated in silico design strategy for the discovery of novel STING agonists. **Conclusions**: The identified compounds represent promising candidates for subsequent experimental validation and may support the development of nitrogen heterocycle-based STING agonists for antitumor applications.

## 1. Introduction

STING is a pivotal adaptor protein in innate immunity, primarily localized on the endoplasmic reticulum (ER). Upon sensing cytosolic DNA, STING undergoes conformational changes and oligomerization, thereby activating downstream signaling pathways. The cyclic GMP–AMP synthase (cGAS)–STING axis constitutes the core mechanism for cytosolic DNA recognition in mammalian cells. Specifically, cGAS senses cytoplasmic DNA and catalyzes the synthesis of 2′3′-cyclic GMP–AMP (2′3′-cGAMP), which binds to and activates STING, ultimately inducing the production of type I interferons (IFNs) and multiple pro-inflammatory cytokines [1,2,3,4,5]. This pathway plays a critical role in antiviral defense, antitumor immunity, and the initiation of adaptive immune responses [6,7,8,9,10]. Consequently, dysregulation of STING signaling is closely associated with infectious diseases, malignancies, and autoimmune disorders, highlighting its potential as a therapeutic target.

Early efforts to develop STING agonists primarily focused on cyclic dinucleotides (CDNs). Among these, the endogenous ligand 2′3′-cGAMP was the first identified natural agonist [11,12], followed by the development of analogs such as ADU-S100 [13,14], MK-1454 [15], and SB11285 [16]. Although CDNs are highly active in vitro and bind strongly, their large size, poor membrane permeability, susceptibility to enzymatic degradation, and short in vivo half-life limit their clinical use [17,18]. Moreover, several CDN candidates have demonstrated unsatisfactory efficacy in clinical trials, whether administered as monotherapy or in combination with immune checkpoint inhibitors [14,19,20,21]. These limitations have prompted the exploration of non-nucleotide agonists (non-CDNs) with improved drug-like properties.

Among non-CDN STING agonists, ABZI derivatives have emerged as particularly promising. Ramanjulu et al. first reported the dimeric ABZI (diABZI) in 2018, which achieved high-affinity binding by spanning the STING dimer pocket and induced complete and durable tumor regression in syngeneic mouse models via intravenous administration, overcoming the intratumoral delivery limitation of CDNs [22]. Nevertheless, diABZI compounds generally require systemic administration and exhibit limited oral bioavailability and potential systemic inflammatory risk [23]. In 2020, Xi et al. reported a series of ABZI derivatives that robustly induced IFN-β, CXCL10, and IL-6 production in vitro. These compounds also demonstrated favorable antitumor activity in mouse models and were evaluated comprehensively for their pharmacokinetic properties [24]. Despite these advances, systemic administration remained necessary, and inter-allelic differences among human STING variants pose translational challenges [25]. Further optimization by Song et al. led to the synthesis of Triazole-40, which maintained high activity, exhibited over 20-fold improved aqueous solubility compared with earlier analogs, and stably bound multiple human STING alleles, highlighting enhanced drug development potential [26]. Nonetheless, significant potency discrepancies between human and murine STING underscore the need for careful cross-species model selection [26]. In addition, ABZI compounds have demonstrated utility beyond oncology; for example, diABZI-4 administered intranasally effectively inhibited SARS-CoV-2 replication and conferred protection in K18-ACE2 transgenic mice [26]. Collectively, ABZI derivatives combine potent STING agonistic activity with favorable drug-like characteristics, yet challenges including oral bioavailability, cross-species potency differences, and systemic inflammatory risk remain, offering opportunities for further structural optimization and delivery strategy development [23,26,27].

In this work, a 3D quantitative structure–activity relationship (3D-QSAR) model was developed to investigate the structure–activity relationships of ABZI derivatives targeting STING. A dataset of 109 ABZI analogs with reported in vitro agonistic activities was collected from the literature [24,28,29,30,31] and used to construct a Topomer CoMFA model, enabling quantitative evaluation of key steric and electrostatic features governing biological activity [32]. Key structural features were extracted from the 3D-QSAR contour maps and used to guide subsequent molecular design. Subsequently, a fragment-based molecular growth strategy was employed to design new candidate compounds, followed by molecular docking to predict potential binding modes, molecular dynamics simulations to assess complex stability, and MM-PBSA calculations to estimate binding free energies. Five prioritized compounds were identified, among which compounds 1–3 exhibited favorable predicted affinity, Caco-2 permeability, microsomal metabolic stability, and toxicity profiles. Additionally, computer-aided synthetic planning (CASP) using AI tools provided feasible synthetic routes for the selected compounds, supporting subsequent experimental synthesis and activity validation. This study provides a framework to support the rational design of ABZI-derived non-CDNs STING agonists with potential antitumor applications.

## 2. Result and Discussion

### 2.1. 3D-QSAR Model Construction and Validation

**Figure 1:**
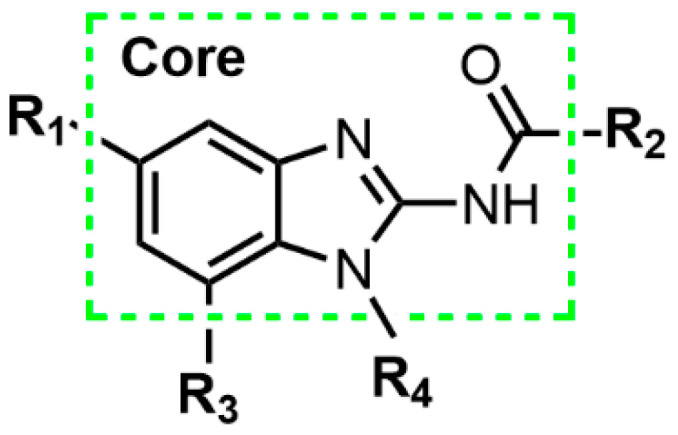
The common structure of ABZI agonists (the part inside the dotted line box is the core group, and R1–R4 represent substituents).

Based on 89 training compounds, a 3D-QSAR model for ABZI derivatives was constructed using the Topomer CoMFA approach. The fragmentation scheme applied to the first compound in the training set is illustrated in Figure 1, which ensures that the designed agonists retain the core scaffold while capturing the influence of various R-group substitutions on biological activity. To quantitatively evaluate model performance, statistical parameters were calculated using both leave-one-out cross-validation and non-cross-validation methods. The optimal number of components (ONC) was identified as 3, resulting in a cross-validated correlation coefficient of 0.55, a conventional correlation coefficient of 0.72. These metrics satisfy the commonly accepted standards for 3D-QSAR modeling, indicating that the model captures meaningful structure–activity relationships within the training set.

**Figure 2:**
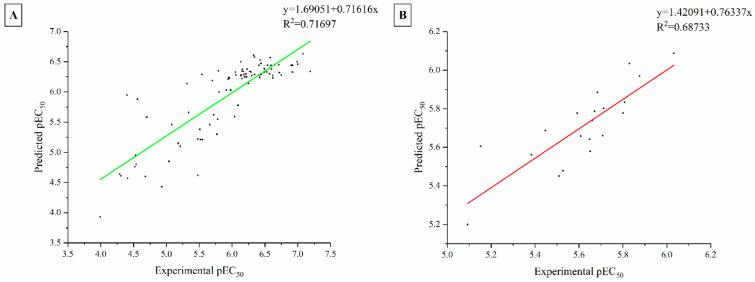
The linear regression relationship between experimental and predicted activity for training set (**A**) and test set (**B**). (The correlation coefficients of are 0.7170 and 0.6873 respectively.)

To further evaluate predictive reliability the model, an external test set comprising 20 compounds (compound 90–109, Table S1) was employed. The external predictive correlation coefficient ($R_{external}^{2}$) was 0.69, suggesting reasonable generalization. The correlation between experimental and predicted pEC_50_ values for both the training and test sets is depicted in Figure 2, providing a clear visualization of the model’s ability to capture the activity trends of ABZI derivatives. These results demonstrate that the Topomer CoMFA model’s ability to reproduce overall activity trends of ABZI derivatives.

Finally, Y-randomization tests were further performed to validate the robustness of the model. In this procedure, the independent variables were kept unchanged, while the dependent variable (pEC_50_) was shuffled 10 times. New models were then reconstructed. Compared to the original model, all the randomized models yielded markedly lower q^2^ and r^2^ values (Table 1), which indicates that the constructed Topomer CoMFA model is robust and free from spurious correlations.

### 2.2. COMFA-Based Structure–Activity Relationship Analysis

**Figure 3:**
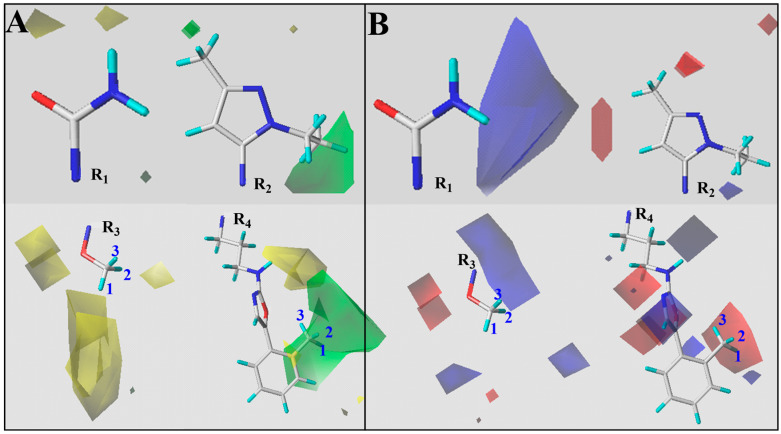
Contour Maps of Steric (**A**) and Electrostatic Fields (**B**) for R1-R4 Substituents of Reference Compound D59. For Contour Maps of Steric: green regions indicate where bulky substituents increase biological activity, while yellow regions indicate where less bulky substituents favor higher activity; For Electrostatic Fields: blue regions favor positively charged groups, and red regions favor electronegative groups for improved agonist activity.

The quantitative structure–activity relationship of ABZIs derivatives was analyzed using the Topomer CoMFA model. Colored contour maps generated from the StDev × Coeff coefficients were employed to visualize the relationship between molecular features and biological activity. The contour maps correlate variations in compound activity with steric and electrostatic fields. Steric contours are represented in green and yellow, while electrostatic contours are shown in blue and red (Figure 3). Green steric regions indicate that increasing bulk at these positions is favorable for activity, whereas yellow regions suggest that steric expansion should be avoided. In electrostatic maps, blue regions are favorable for electronegative substituents, while red regions are favorable for electropositive groups to enhance activity. Here, it was also compound D59 used as a reference, the analysis of substituents at each position is as follows:

R1 substituent: The steric map shows limited green regions, indicating that increasing bulk at this position is unfavorable. This observation is supported by the comparison of compounds 3 and D5, and compounds 4 and 14b with their respective pIC_50_ values.

R2 substituent: A small green area is present near the ethyl group on the pyrazole ring, suggesting that introducing small functional groups or substituting the ethyl group may enhance activity.

R3 substituent: Large green polyhedral regions are observed below the fragment, indicating that introducing bulk at this position is beneficial. Experimental data of compounds 24b vs. 16c and 14f vs. 24c confirms this conclusion.

R4 substituent: The terminal methyl group on the phenyl ring is located in a large green area, and electrostatic analysis indicates that incorporating electronegative groups at this position could further enhance activity.

The steric and electrostatic field analysis suggests that R3 is suitable for bulk optimization, while the terminal phenyl ring of R4 is appropriate for the introduction of electronegative functional groups. Based on these insights, newly designed ABZIs derivatives will focus on fragment growth at the R3 position and the introduction of electronegative groups at the R4 terminal phenyl ring to improve biological activity.

In this work, the R3 site was diversified using the fragment-growing function in MOE, while the R4 site was substituted with groups such as amino, nitro, and halogens. The built-in Linker fragment library in MOE covers a wide chemical space, providing structural diversity for fragment growth. The newly generated compounds, together with seven template molecules, gave a total of 94,850 candidates. Their binding affinity to the STING protein was first evaluated using the London dG scoring function, which was applied during the fragment growth process. Finally, a total of 1030 compounds with binding energies below −15.00 kcal/mol were selected for further analysis [33], and their London dG scoring and molecular docking are shown in Table S2.

### 2.3. Molecular Docking

Molecular docking was performed on the 1030 newly designed compounds obtained from Section 3.2. Among them, 773 showed better binding energies than the reference compound D59 (−8.47 kcal/mol), suggesting stronger affinity for STING. This outcome also reinforces the reliability of the CoMFA model established in Section 3.1 and Section 3.2.

To identify the most promising candidates, the top 50 compounds based on docking scores were further analyzed, and 10 representative molecules were selected for detailed evaluation (Table 2). These compounds exhibited notably stronger binding to STING than D59, with CoMFA-predicted pEC_50_ values ranging from 5.00 to 6.00.

**Figure 4:**
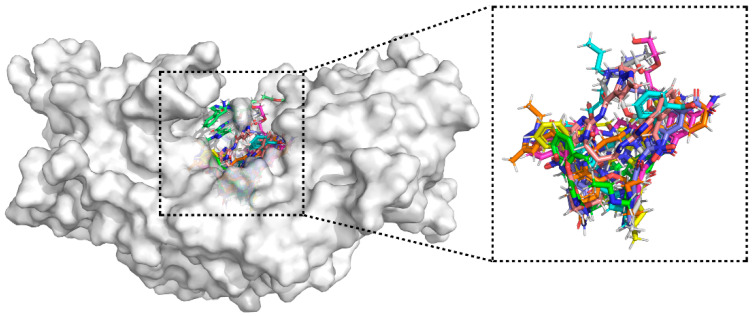
Surface representation of STING with ten compounds bound in the ligand-binding pocket. Each color represents a different compound.

Binding mode analysis showed that these 10 compounds consistently occupied the STING active pocket and formed extensive non-covalent interactions with surrounding residues (Figure 4). Notably, nine of them introduced negatively charged substituents (–NH_2_, –NO_2_, or halogens) at the R4 position (Table 3). These groups established stable hydrogen bonds with key residues and enhanced hydrophobic contacts, leading to improved binding stability between the ligands and STING.

### 2.4. Multi-Step Binding Free Energy Calculation

In this study, the 10 ABZI derivatives selected in Section 3.3, together with the reference compound D59, were subjected to 100 ns molecular dynamics (MD) simulations in complex with the STING protein, and the binding free energies were also calculated from the trajectories. As shown in Table 2, four compounds (M1, M11, M13, and M44) exhibited binding free energies lower than that of D59 (−38.88 kcal/mol), with M44 showing a reduction of 4.59 kcal/mol relative to D59, indicating stronger binding affinity to STING.

**Figure 5:**
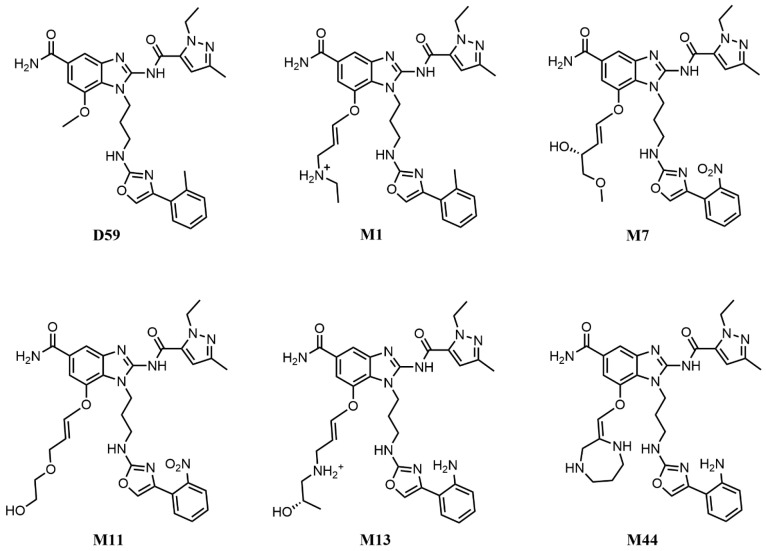
Structures of six STING agonists.

**Figure 6:**
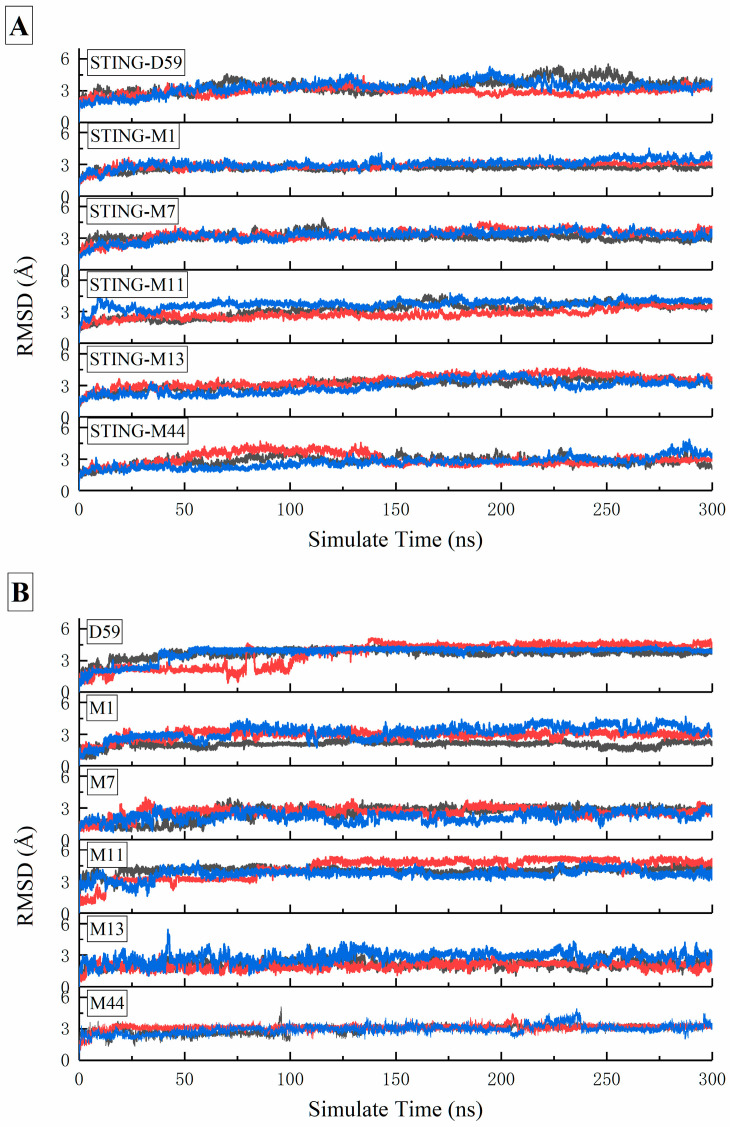
RMSD values of (**A**) the complexes and (**B**) ligands from three parallel 300 ns molecular dynamics simulations (black, red, and blue).

To address the potential low accuracy associated with using a single short MD simulation for MM-PBSA calculations, we extended the analysis for the four selected complexes (STING-M1, STING-M11, STING-M13, and STING-M44) along with the reference STING-D59 and STING-M7. Each system underwent four independent 300 ns MD simulations in parallel, with the final binding free energies reported as the average across these replicates. This multi-replicate approach was specifically implemented to ensure statistical reliability and to effectively mitigate sampling variability inherent in shorter or single-trajectory simulations. Although M7 exhibited a slightly higher binding free energy than D59 (0.11 kcal/mol), it demonstrated a superior docking score and a more favorable binding mode. Therefore, STING-M7 was also included (the structures of these six compounds were presented in Figure 5). The calculated root-mean-square deviation (RMSD), listed in Figure 6, showed that all systems converged and stabilized around 3 Å, indicating that the complexes maintained stable conformations throughout the 300 ns simulations.

Finally, for each simulation, one frame was saved every 20 ps during the last 20 ns of the equilibrated trajectory, giving 1000 frames per system for subsequent binding free energy calculations. As summarized in Table 4, the binding free energies calculated from four independent 300 ns MD simulations are highly reproducible, with standard errors of 0.86–1.88 kcal/mol across all compounds. In particular, M13 (−43.30 ± 0.98 kcal/mol), M11 (−34.66 ± 0.95 kcal/mol), and M1 (−37.62 ± 0.86 kcal/mol) display SE < 1 kcal/mol, demonstrating robust convergence and reliable statistical meaning of the results. Compared to the reference compound D59 (−35.78 kcal/mol), all derivatives except M11 display more favorable binding free energies. Notably, M13 and M44, exhibited reductions of 7.5 kcal/mol and 6.7 kcal/mol respectively, demonstrating exceptionally high binding affinity to the STING protein.

### 2.5. Binding Mode

**Figure 7:**
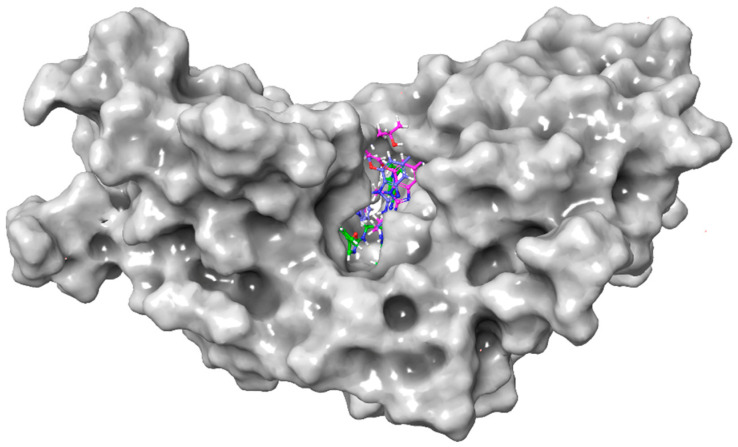
Surface representation of STING and structural superposition of four ligands after MD simulations.

**Figure 8:**
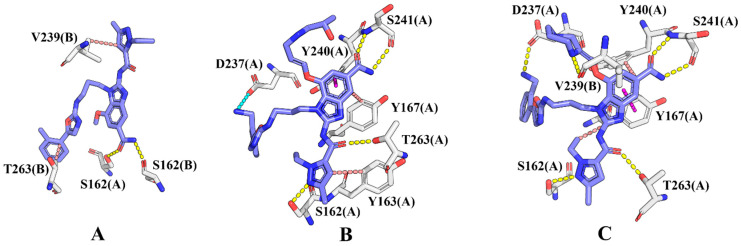
Binding modes of the STING protein complex systems. (**A**) STING-D59, (**B**) STING-M13 complex and (**C**) STING-M44. Dashed lines of different colors indicate specific intermolecular interactions: yellow for hydrogen bonds, cyan for salt bridges, magenta for π-π stacking interactions, and salmon for hydrophobic interactions.

To further elucidate the binding mechanisms of the candidate ABZI derivatives with STING, representative conformations from the equilibrated MD trajectories were analyzed in combination with hydrogen bond occupancy and per-residue decomposition of the MM-PBSA energies. As shown in Figure 7**,** the reference compound D59 as well as the derivatives M13 and M44 were stably embedded in the STING binding pocket while retaining the conserved ABZI scaffold. The central amide group, serving as a key pharmacophore, formed conserved hydrogen bonds with loop-region residues in the binding site (Figure 8). However, compared with D59, which formed only two primary hydrogen bonds with SER162(A) and SER162(B) (with the highest occupancy of merely 59.62%), M13 and M44 adopted more extended conformations due to the substitutions introduced at the R3 and R4 positions (Table 5). This spatial rearrangement facilitated the formation of a more extensive and stable noncovalent interaction network with surrounding residues.

Specifically, M13 not only maintained the core amide–SER interaction but also established a strong hydrogen bond between the N5 atom of its 1H-pyrazole group and SER162(A)-OG, with an occupancy of 79.71%. In addition, the amide carbonyl formed a hydrogen bond with SER241(A)-N (64.52% occupancy), together with a hydrogen bond to THR263(A) (50.67% occupancy), constituting a stable “Ser162–Ser241–Thr263” three-point hydrogen-bonding network. Moreover, the electron-rich –NH_2_ group introduced at the R4 position of M13 formed a stable salt bridge with Asp237(A), further extending the ligand conformation and enhancing binding specificity toward the pocket residues.

M44 also preserved multiple hydrogen bonds with SER162(A), SER241(A), and THR263(A), each with an occupancy greater than 60%. In addition, the piperazine substituent introduced a new hydrogen bond donor, forming a unique cross-chain hydrogen bond with VAL239(B)-O, albeit with a lower occupancy of 21.04%. Furthermore, the bulky R3 substituent of M44 enlarged the hydrophobic contact surface, strengthening van der Waals and π–π stacking interactions with Tyr167(A) and surrounding residues, while the amino group at the R4 position engaged in a weaker hydrogen bond with Asp237(A).

**Figure 9:**
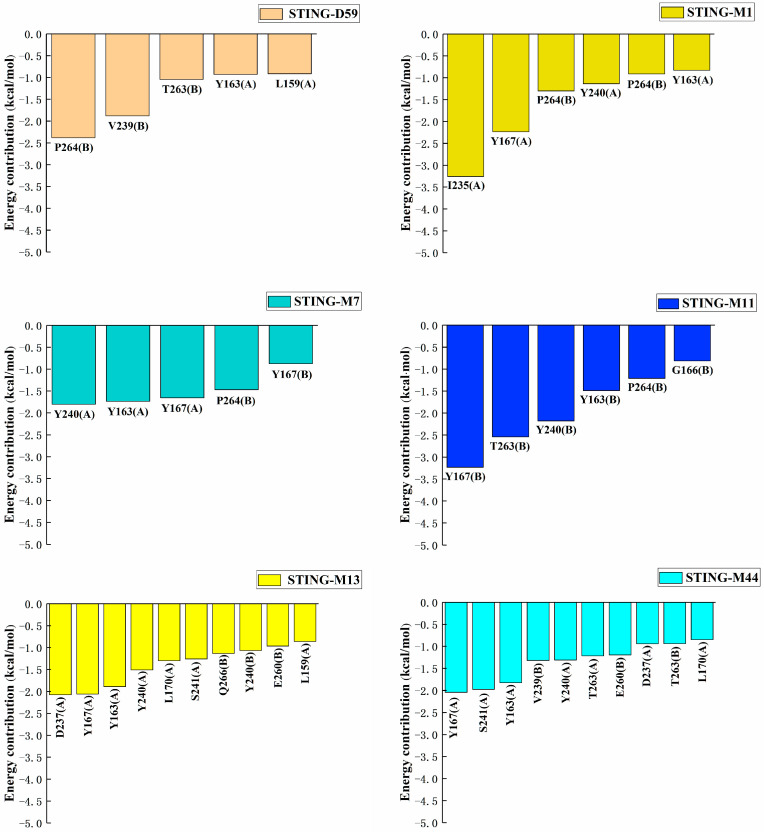
Residue energy contributions to the binding free energy (only residues with an energy contribution magnitude greater than 0.8 kcal/mol are shown).

Therefore, the amide groups of both M13 and M44 formed stable hydrogen bonds with the Ser241 residue located in the loop region of chain A at the upper part of the STING active pocket, while their 1H-pyrazole moieties established hydrogen bond interactions with Ser162 and other residues at the lower part of the pocket. In addition, the benzimidazole rings of both compounds engaged in hydrophobic interactions and π–π stacking with Tyr167(A), thereby firmly anchoring the ligands within the active pocket. The residue-based free energy decomposition analysis (Figure 9) further revealed that these key residues, including Ser162, Tyr167, and Ser241, made substantial contributions to the binding free energy and played central roles in maintaining the stability of ABZIs–STING interactions.

### 2.6. ADMET Evaluation

To evaluate the drug-likeness of the designed compounds, ADMET predictions were performed for the candidate compounds M13 and M44, as well as the reference compound D59 [34]. In terms of physicochemical properties, parameters such as aqueous solubility (Log S) and the octanol/water partition coefficient (Log P) not only determine solubility, membrane permeability, and potency, but also exert a crucial impact on pharmacokinetic (PK) and pharmacodynamic (PD) characteristics. Since physicochemical features are largely determined by chemical structure, the introduction of carbon chains or cyclic moieties at the R3 position enhanced hydrophobic and van der Waals interactions of M13 and M44 within the binding pocket, thereby strengthening their binding affinity and specificity toward STING. However, the increase in molecular weight generally leads to decreased solubility. This drawback was partially compensated in M13 and M44 by introducing hydrophilic groups such as ether, hydroxyl, or quaternary ammonium substituents at the R3 position, which improved their Log S and Log P values compared with the reference compound D59.

Regarding drug distribution (Table 6), the predicted blood–brain barrier (BBB) permeability of the reference compound D59 was poor, suggesting its suitability for peripheral targeting. By contrast, certain designed molecules (e.g., M13) showed favorable BBB penetration, implying potential as central nervous system (CNS)-targeted agents. Plasma protein binding (PPB) analysis indicated that D59 exhibits strong binding to plasma proteins, which may lower the free drug concentration in vivo and reduce oral bioavailability. In comparison, M13 and M44 displayed more favorable PPB profiles.

Toxicity assessment revealed that cardiotoxicity remains a potential concern. The human ether-a-go-go related gene (hERG) channel blockade, a key predictor of drug-induced cardiotoxicity, indicated that both D59 and M13 present relatively high risks, which may pose challenges for their further developability and therefore warrant careful consideration in lead optimization, as well as subsequent in vitro validation of cardiac safety. For skin sensitization, all tested molecules contained alerting structures such as 1H-benzo[d]imidazol-2-amine, which may trigger hypersensitivity reactions; moreover, the amino substituents introduced in M13 and M44 could further increase sensitization risk. These structural alerts suggest that future optimization should focus on scaffold modification or substituent tuning to mitigate sensitization liabilities while preserving STING activity. In cytotoxicity prediction assays against A549 (lung adenocarcinoma) and normal cells, the overall toxicity levels of the candidate compounds were lower than that of D59, indicating a comparatively favorable cellular safety profile and supporting their prioritization as in silico starting points rather than fully validated leads.

### 2.7. Retrosynthetic Analysis

Structurally, the compounds consist of 4-, 5-, or 6-ring systems with moderate complexity, lacking excessive fused or bridged rings that are known to elevate synthetic challenges due to increased stereochemical and regioselective demands. Additionally, all four compounds exhibited zero violations of Lipinski’s Rule of Five, confirming their drug-like properties with balanced physicochemical profiles and no evident structural liabilities such as atomic diversity extremes or atypical functional groups. This enhances their post-synthesis application potential in drug development.

To evaluate the synthetic accessibility of the identified compounds, we employed a combination of established metrics, starting with the widely used Synthetic Accessibility Score (SAscore), a molecular structure-based heuristic [34]. As shown in Table 6, the SAscore values for these four compounds are all <6, indicating they are all relatively easy to synthesize. Complementing this, we utilized DeepSA, a deep learning-driven chemical language model trained on over 3.5 million molecules using natural language processing techniques to predict synthesis accessibility [35]. Table S3 presents the DeepSA predictions: ES probabilities spanned 0.77 to 0.997, while HS probabilities were consistently low, spanning 0.0029 to 0.222. All HS values were well below the model’s thresholds of 0.47 for ES/HS balance and 0.5 for definitive HS classification. These results collectively indicate low synthetic difficulty, aligning with DeepSA’s high discriminative performance (AUROC of 89.6% in validation studies).

To assess overall synthetic accessibility based on molecular structure, we applied the Retrosynthetic Accessibility Score (RAscore), a machine learning model trained on AI-driven retrosynthesis data to predict synthesizability by estimating whether a viable synthetic pathway can be identified via computer-aided synthesis planning [36]. As shown in Table S4, most compounds exhibited high RAscores (0.59–0.87), indicating favorable synthetic feasibility, except for M11 (0.33), which suggests higher potential synthesis challenges.

Building on these predictions, we further validated feasibility by generating retrosynthetic routes using ChemAIRS (https://www.chemiscal.com/), an AI-powered retrosynthesis platform developed by Chemical. AI that leverages machine learning and expert chemical rules to generate diverse, efficient pathways by analyzing thousands of potential routes. The highest-ranked route for each compound was evaluated, with that of M13 serving as a representative example and selected as the primary synthesis scheme for this study (Figure S1). These assessments collectively support the compounds’ practical viability for experimental synthesis in drug development.

## 3. Materials and Method

### 3.1. Data Sets and Structure Alignment

In this work, a total of reported 109 ABZI derivatives reported were collected as the dataset for 3D-QSAR modeling, and their chemical structures and biological activities are summarized in Table S1. The EC_50_ values of the collected compounds were within the range of 0.065 to 51.07 μM. For compatibility with the 3D-QSAR model, EC_50_ values were converted to pEC_50_ (−logEC_50_), with values ranging from 3.991 to 7.187.

All compounds were preprocessed using SYBYL-X v2.0. Molecular 3D conformations were optimized with the Tripos force field, and Gasteiger–Hückel charges were assigned. Energy minimization was performed using the Powell gradient algorithm, with a maximum of 1000 iterations and an energy convergence criterion of 0.001 kcal/mol/Å^2^. To ensure conformational reliability, the lowest-energy conformation of each molecule was selected as the representative structure for subsequent modeling.

The selection of a template compound is critical for the accuracy of 3D-QSAR alignment. In this study, the most potent compound, D59, was chosen as the template, and all other molecules were aligned to its core scaffold. The dataset was randomly divided into a training set (89) for model construction and a test set (20) for external validation.

### 3.2. Topomer CoMFA Model Construction

The 3D-QSAR model was constructed using the Topomer CoMFA approach implemented in SYBYL-X v2.0 [37]. Topomer CoMFA is a fragment-based method in which molecules are divided into a core scaffold and R-group substituents. Conformations of the fragments are automatically generated and aligned according to empirical rules, thereby creating a three-dimensional lattice encompassing all compounds in the training set. The lattice spacing was set to 2.0 Å, and an sp^3^-hybridized carbon atom probe was applied to calculate steric and electrostatic field energies around the molecules. The resulting descriptors were used as independent variables, while the pEC_50_ values served as dependent variables for regression analysis using the partial least squares (PLS) method [38].

The construction of the Topomer CoMFA model was based on the optimal number of principal components, which was determined through cross-validation. The predictive quality of the established model was assessed using both cross-validated and non-cross-validated approaches, yielding the cross-validation coefficient (q^2^) and the correlation coefficient (r^2^), respectively. To further evaluate the robustness of the model, Y-randomization testing was performed: while keeping the independent variables (X) unchanged, the dependent variable (pEC_50_) was randomly permuted 10 times and the models were reconstructed. In all randomized cases, the resulting q^2^ and r^2^ values were markedly lower than those of the original model, indicating that the Topomer CoMFA model possessed reliable predictive capability. The validated 3D-QSAR model further enabled the interpretation of structure–activity relationships of ABZI derivatives through contour map analysis and facilitated the prediction of their bioactivity values.

### 3.3. Fragment Growing

Based on the structure–activity relationships revealed by the Topomer CoMFA model constructed in Section 3.2, a fragment-based growth strategy was employed to optimize the ABZI scaffold and design potential derivatives with enhanced bioactivity. The most active compound, D59, was selected as the template, with the R3 and R4 positions designated as primary modification sites. Fragment growth was performed using the Linker fragment library integrated in MOE v2019 (Chemical Computing Group, Montreal, QC, Canada) [39], which encompasses a variety of common functional groups and structural motifs, thereby enabling coverage of a broad chemical space.

For the newly generated molecules, preliminary evaluation of their binding affinity toward the STING protein was conducted using the London dG [40] scoring function. London dG is an empirical scoring function that estimates the relative binding free energy between small molecules and their receptor, allowing rapid assessment of potential interactions. In the present study, the obtained scores were used to prioritize candidate compounds and guide subsequent molecular docking and molecular dynamics simulations.

### 3.4. Molecular Docking

To investigate the binding affinity and interaction patterns between the newly designed ABZI derivatives and STING, molecular docking simulations were performed using MOE v2019 [39]. Crystal structures of STING complexed with ABZI ligands (PDB IDs: 6DXL) [22] were obtained from the RCSB Protein Data Bank [41]. All protein and ligand structures were prepared and minimized using the Amber10:EHT force field in MOE prior to docking. Missing amino acid residues in the protein were completed using QuickPrep (MOE), followed by protonation via Protonate3D, and energy minimization with an RMS gradient of 0.1 kcal/mol/Å^2^. Ligands were prepared by adding hydrogen atoms, computing partial charges, and performing energy minimization under the same parameters.

The prepared protein structures were designated as receptors and the ligands as docking partners, with the binding site defined by the location of the co-crystallized ligand. Docking was conducted in two stages: placement and refinement. During the placement stage, the Triangle Matcher method was used to generate initial poses, which were scored by the London dG function, and the top 50 poses were selected for further refinement. In the refinement stage, a semi-flexible docking protocol was employed, keeping the protein rigid while allowing ligand flexibility, and the GBVI/WSA dG scoring function was used to evaluate the binding affinity of each pose. The final docking results were reported as the best-scoring conformations.

### 3.5. Molecular Dynamics Simulation and Binding Free Energy Calculation

Molecular dynamics (MD) simulations were performed using Amber18. Topology and initial coordinates of all the system were prepared with the tleap module of AmberTools18. The ff14SB force filed [42] was applied to the protein, while the ligand was described using GAFF [43], with missing parameters generated via parmchk2. The complex was solvated in an octahedral TIP3P water box, ensuring a minimum distance of 12 Å between any solute atom and the box edge, and counterions (Na^+^/Cl^−^) were added to neutralize the system.

Energy minimization was conducted in two stages. In the first stage, a Cartesian restraint of 500 kcal/mol/Å^2^ was applied to the protein and ligand atoms, and the solvent molecules were minimized for 10,000 steps (first 5000 steps by steepest descent, followed by 5000 steps of conjugate gradient). In the second stage, all restraints were removed, and the entire system underwent an 10,000-step unrestrained minimization.

The minimized system was gradually heated from 0 K to 300 K under the NVT ensemble, with restraints on the protein backbone atoms to prevent unrealistic conformational changes, using the Langevin thermostat. Pre-equilibration was then performed under NPT conditions (1 atm, 300 K) for 2 ns. The production phase consisted of three independent 300 ns MD simulations under the same NPT conditions, each initiated with a different random seed to ensure independent trajectories. A time step of 2 fs was used, and all bonds involving hydrogen atoms were constrained using the SHAKE algorithm [44]. Non-bonded interactions were truncated at 10 Å, and long-range electrostatics were treated using the Particle Mesh Ewald method [45]. Atomic coordinates were saved every 50 ps for subsequent analysis.

Trajectory analysis was performed using Cpptraj, and the RMSD of the system was calculated to assess equilibration. To evaluate the binding affinity between receptor and ligand, Molecular Mechanics/Poisson–Boltzmann Surface Area (MM-PBSA) calculations were carried out using MMPBSA.py [46,47]. The average binding free energy was computed over 2000 frames extracted from the last 20 ns of stable trajectory. The binding free energy $\Delta G_{bind}$ could be calculated from:

(1) $$\Delta G_{bind}=G_{complex}-(G_{receptor}+G_{ligand})$$

Subsequently, per-residue decomposition analysis was performed for the last 20 ns of the trajectory to identify key amino acid residues contributing significantly to the binding free energy and to elucidate their roles in ligand–receptor interactions.

### 3.6. Retrosynthetic Analysis

To reduce trial costs and shorten the synthesis cycle for target compounds, this work used the online server ChemAirs (pro.chemiscal.com/home) to assess the synthetic difficulty of candidate compounds and predict synthetic routes. The server uses the SA score (1–10, with higher scores indicating greater synthetic difficulty) to quantify synthetic accessibility. The retrosynthetic analysis based on chemical reaction patterns, uses chain scission to gradually decompose the target molecule into intermediates and commercially available precursors. The server’s default parameters were used throughout the analysis. Retrosynthetic recommendations were screened based on synthetic difficulty and raw material costs to determine the optimal synthetic strategy.

## 4. Conclusions

In this work, an integrated computational framework combining 3D-QSAR modeling, fragment-based molecular design, molecular docking, molecular dynamics simulations, binding free energy calculations, and ADMET prediction was applied to the rational optimization of ABZI-derived STING agonists, identifying steric expansion at the R3 position and the introduction of electronegative substituents at the R4 terminal phenyl ring as favorable strategies for enhancing STING agonistic activity. Guided by these findings, a large virtual compound library was generated and systematically screened, leading to the identification of several high-affinity candidates.

M13 and M44 exhibited markedly enhanced binding affinity compared with the reference compound D59, as confirmed by extensive molecular dynamics simulations and MM-PBSA calculations. These compounds formed stable and extensive noncovalent interaction networks with key residues in the STING binding pocket, including conserved hydrogen bonds with Ser162 and Ser241, as well as strengthened hydrophobic and π–π interactions with Tyr167. ADMET analyses further indicated that M13 and M44 possess improved physicochemical and pharmacokinetic properties, while maintaining acceptable safety profiles, highlighting their potential as lead compounds for further development. In addition, AI-assisted synthetic route prediction provided practical guidance for their experimental synthesis and biological evaluation.

Although this is an in silico discovery study, the identified computational leads may offer valuable structural insights and inspiration for non-CDN STING agonist research. However, synthesis of these novel ABZI derivatives is currently challenging due to limited access to key intermediates; we are actively trying to obtain these intermediates, exploring viable synthetic routes, and planning to experimentally validate the top candidates in subsequent work.

## Figures and Tables

**Table 1:** Y-randomization validation results of the Topomer CoMFA model.

Iteration	q^2^	r^2^
Random 1	−0.0026	0.2172
Random 2	−0.0455	−0.0622
Random 3	−0.0387	0.0885
Random 4	−0.0235	−0.1351
Random 5	−0.0445	−0.0147
Random 6	0.0100	−0.2098
Random 7	−0.0386	0.0363
Random 8	−0.0495	0.0368
Random 9	−0.0454	0.0463
Random 10	−0.0405	0.0827

**Table 2:** Docking scores of the top 50 compounds based on the London dG scoring function, fragment growth scores, and protein–ligand binding free energies obtained from 100 ns molecular dynamics simulations. Na denotes unavailable values.

Id	Compound Name	Pred (pEC_50_)	London dG (kcal/mol)	Docking Scores (kcal/mol)	Binding Free Energy PB (kcal/mol)
1	D59	6.34	Na	−8.47	−38.88 ± 0.13
2	O4H2	6.41	Na	−8.24	Na
3	O4O2	6.24	Na	−8.75	Na
4	O4B	6.43	Na	−7.99	Na
5	O4C	6.42	Na	−8.82	Na
6	O4F	6.40	Na	−8.29	Na
7	O4CH	6.49	Na	−8.34	Na
8	M1	6.33	−15.10	−9.83	−40.57 ± 0.15
9	M3	6.71	−15.18	−9.68	−34.14 ± 0.20
10	M6	6.50	−15.35	−9.65	−30.60 ± 0.34
11	M7	6.09	−16.36	−9.63	−38.77 ± 0.18
12	M11	6.31	−16.41	−9.58	−40.31 ± 0.26
13	M13	6.57	−15.15	−9.57	−41.15 ± 0.18
20	M44	5.98	−15.16	−9.36	−43.47 ± 0.18

**Table 3:** Structures of the design compound of the present study.

Id	Compound Name	Compound Structure	R3	R4
1	D59	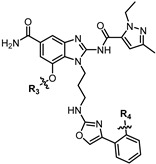	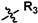	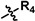
2	O4H2		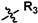	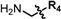
3	O4O2		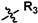	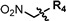
4	O4B		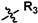	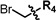
5	O4C		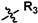	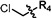
6	O4F		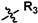	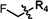
7	O4CH		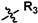	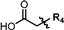
8	M1		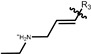	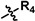
10	M3		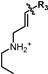	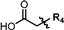
11	M6		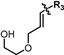	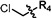
12	M7		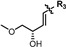	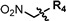
13	M11		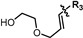	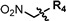
14	M13		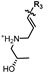	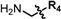
15	M16		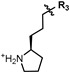	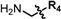
16	M20		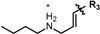	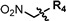
17	M23		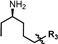	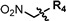
18	M25		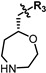	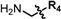
19	M38		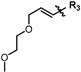	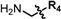
20	M44		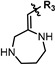	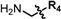

**Table 4:** Binding free energies obtained from three parallel 300 ns MD simulations (kcal/mol).

	Parallel 1	Parallel 2	Parallel 3	Parallel 4	Total G_bind_
D59	−32.02	−40.87	−34.30	−35.91	−35.78 ± 1.88
M1	−39.18	−35.42	−37.10	−38.78	−37.62 ± 0.86
M7	−34.95	−41.16	−39.92	−40.66	−39.17 ± 1.43
M11	−34.64	−35.83	−31.98	−36.20	−34.66 ± 0.95
M13	−41.01	−45.78	−43.27	−43.14	−43.30 ± 0.98
M44	−42.89	−39.60	−45.77	−41.77	−42.51 ± 1.28

**Table 5:** Hydrogen bonds formed in these systems.

System	Acceptor	Donor	Hydrogen Occupancy	AvgDist (Å)	AvgAng (°)
STING-M13	Lig-N5	SER162(A)-OG	79.71%	2.83	163.57
	Lig-O3	SER241(A)-N	64.52%	2.87	160.81
	Lig-O1	THR263(A)-OG1	50.67%	2.73	156.40
	SER241(A)-O	Lig-N6	41.43%	2.88	162.17
STING-M44	Lig-N5	SER162(A)-OG	69.52%	2.84	163.13
	Lig-O1	THR263(A)-OG1	67.27%	2.70	158.59
	Lig-O3	SER241(A)-N	63.32%	2.85	157.79
	SER241(A)-O	Lig-N6	73.66%	2.85	162.27
	VAL_239(B)-O	Lig-N11	21.04%	2.88	148.53
STING-D59	Lig-O3	SER162(A)-OG	59.62%	2.76	164.39
	SER162(B)-O	Lig-N6	49.43%	2.86	152.50

**Table 6:** The bioavailability and pharmacokinetics prediction.

Properties	D59	M11	M13	M44
MW (g/mol)	556.25	687.28	670.33	667.33
LogS (log_mol/L_)	−4.519	−3.831	−2.964	−2.945
LogP (log_mol/L_)	3.343	2.295	1.859	1.769
SAscore	easy	easy	easy	easy
Lipinski	excellent	excellent	excellent	excellent
Pfizer	excellent	excellent	excellent	excellent
Absorption				
HIA	excellent	excellent	excellent	excellent
PAMPA Permeability	excellent	excellent	excellent	excellent
Distribution				
PPB	poor	poor	excellent	excellent
Volume Distribution	excellent	excellent	excellent	excellent
BBB Penetration	poor	poor	excellent	medium
Metabolism				
CYP1A2 inhibitor/substrate	No/Yes	No	No/Yes	No/Yes
CYP2C19 inhibitor/substrate	No	No	No	No
CYP2C9 inhibitor/substrate	No	YES/No	No	No
CYP2D6 inhibitor/substrate	No/Yes	No	No	No
CYP3A4 inhibitor/substrate	No	YES/No	No	No
CYP2B6 inhibitor/substrate	No	No	No	No
Excretion				
CL_plasma_	excellent	excellent	excellent	excellent
T_1/2_	poor	medium	medium	poor
Toxicity				
hERG Blockers (10 uM)	poor	poor	poor	medium
Skin Sensitization	excellent	poor	medium	medium
AMES Mutagenicity	poor	poor	medium	medium
Carcinogencity	poor	poor	excellent	medium
Hematotoxicity	excellent	medium	excellent	excellent
A549 Cytotoxicity	medium	excellent	excellent	excellent

## Data Availability

The original contributions presented in this study are included in the article and Supplementary Materials. Further inquiries can be directed to the corresponding author.

## Supplementary Materials

The following supporting information can be downloaded at: https://www.mdpi.com/article/10.3390/ph19030387/s1, Table S1: Activity data (pEC_50_) and predicted pEC_50_ values (Pred) of the training set; Table S2: Compound molecular docking score top50; Table S3: The prediction results of DeepSA. HA_num rule_of_five represent the number of heavy atoms, Lipinski’s Rule of Five respectively. ES and HS represent probability values, a molecule requires less than or equal to 10 synthetic steps was labeled as ES, otherwise, if the required step is larger than 10 or can’t be successfully predicted by Retro* was labeled as HS; Table S4: The prediction results of RAscore using XGB model. The values represent synthetic accessibility probability; Figure S1: Predicted synthetic route of compound M13; Figure S2: Details of thepredicted synthetic route for compound M13. References in Supplementary Materials can be found [28,48,49,50,51,52,53,54,55,56,57,58,59,60,61,62,63,64,65,66,67,68,69,70,71,72,73,74,75].
