# Supplementary material for: In Silico Discovery of ABZI Nitrogen Heterocycle STING Agonists via 3D-QSAR, Molecular Dynamics, and AI-Based Synthesis Prediction
Source: Pharmaceuticals (Basel). 2026 Feb 28;19(3):387. doi: 10.3390/ph19030387 (PMC13028874; doi:10.3390/ph19030387)
Supplement: Supplementary file 1 [file pharmaceuticals-19-00387-s001.zip › pharmaceuticals-4140722-supplementary.pdf]

# In Silico Discovery of ABZI Nitrogen Heterocycle STING Agonists via 3D-QSAR, Molecular Dynamics, and AI-Based Synthesis Prediction

**Houcheng Ren [1], Yuhong Jin [2], Baipu Zhao [3], Xiangbing Peng [3], Shan Zhao [4] and Meiting Wang [3,5,*]**

[1] The First Clinical School, Zhengzhou University, Zhengzhou 450001, China
[2] The First Affiliated Hospital, Xinxiang Medical University, Xinxiang 453003, China
[3] Henan International Joint Laboratory of Neural Information Analysis and Drug Intelligent Design, School of Medical Engineering, Xinxiang Medical University, Xinxiang 453003, China
[4] School of Basic Medical Sciences, Xinxiang Medical University, Xinxiang 453003, China
[5] Department of Theoretical Chemistry, Chemical Centre, Lund University, SE-221 00 Lund, Sweden
* Correspondence: meiting.wang@compchem.lu.se

**Table S1.** Activity data ($pEC_{50}$) and predicted $pEC_{50}$ values (Pred) of the training set.

| Id | Compound name | Compound structure | $pEC_{50}$ | Pred |
|---|---|---|---|---|
| 1 | ABZI02_3 | 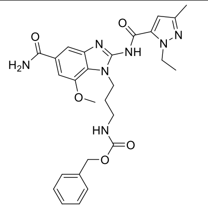 | 6.41 | 6.28 |
| 2 | ABZI_14b | 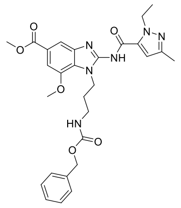 | 4.68 | 4.6 |
| 3 | ABZI_14e | 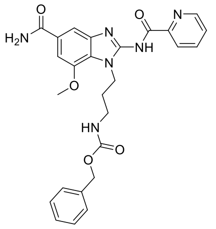 | 5.08 | 5.46 |
| 4 | ABZI_14f | 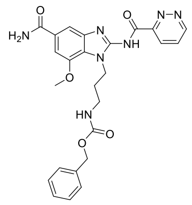 | 5.66 | 5.46 |

| | | | | |
|---|---|---|---|---|
| 5 | ABZI_16a | 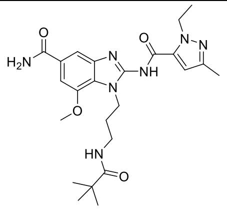 | 4.56 | 5.88 |
| 6 | ABZI_16c | 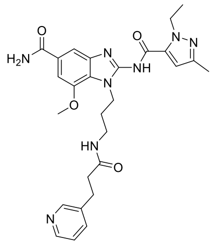 | 4.40 | 5.95 |
| 7 | ABZI_16g | 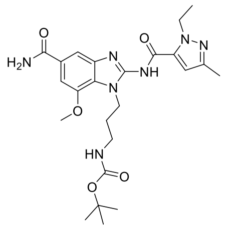 | 5.91 | 6.03 |
| 8 | ABZI_16h | 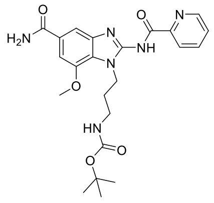 | 5.52 | 5.21 |
| 9 | ABZI_16i | 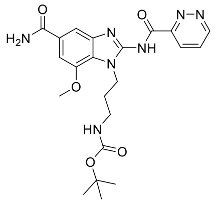 | 5.55 | 5.21 |
| 10 | ABZI_1a | 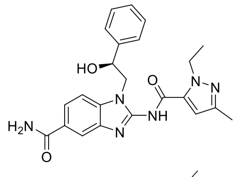 | 3.99 | 3.93 |
| 11 | ABZI_24b | 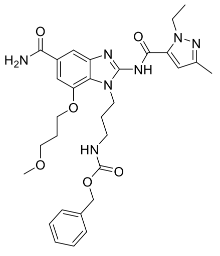 | 6.54 | 6.44 |
| 12 | ABZI_24c | 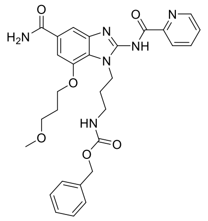 | 5.72 | 5.62 |

| | | | | |
|---|---|---|---|---|
| 13 | ABZI_24e | 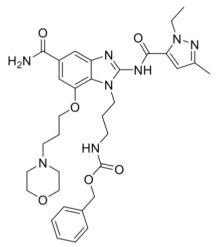 | 5.94 | 6.21 |
| 14 | ABZI_2a | 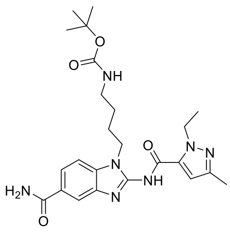 | 5.18 | 5.15 |
| 15 | ABZI_2j | 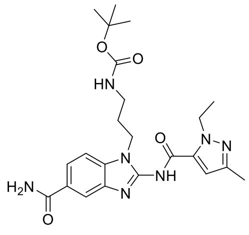 | 4.31 | 4.61 |
| 16 | ABZI_2k | 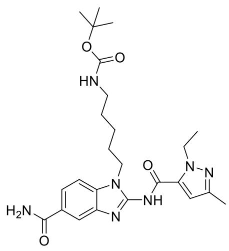 | 5.77 | 5.3 |
| 17 | ABZI_2n | 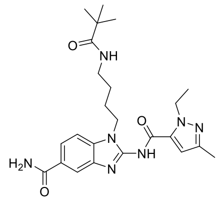 | 4.29 | 4.64 |
| 18 | ABZI_2o | 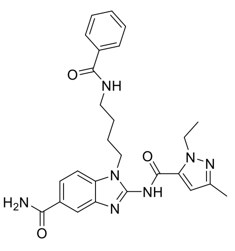 | 4.54 | 4.80 |
| 19 | ABZI_2r | 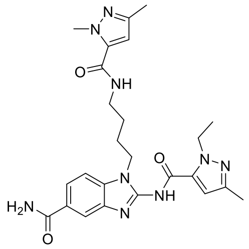 | 4.52 | 4.76 |

| | | | | |
|---|---|---|---|---|
| 20 | ABZI_2s | 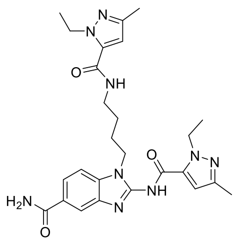 | 5.04 | 4.85 |
| 21 | ABZI_2t | 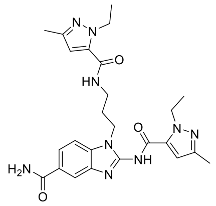 | 4.41 | 4.57 |
| 22 | ABZI_2v | 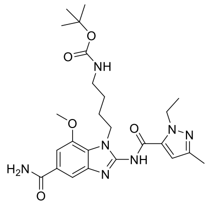 | 6.10 | 5.78 |
| 23 | ABZI_2w | 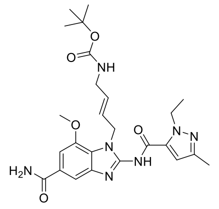 | 5.34 | 5.66 |
| 24 | ABZI_2x | 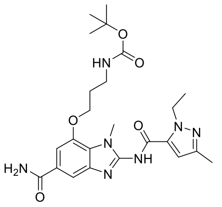 | 5.78 | 5.55 |
| 25 | ABZI_2y | 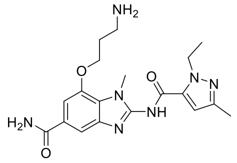 | 4.53 | 4.95 |
| 26 | ABZI_2z | 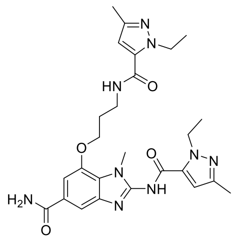 | 5.21 | 5.10 |
| 27 | ABZI_3 | 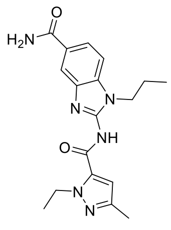 | 5.48 | 4.63 |

| | | | | |
|---|---|---|---|---|
| 28 | ABZI_4 | 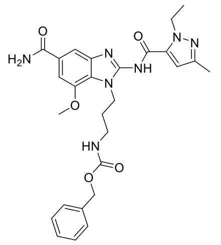 | 6.06 | 6.28 |
| 29 | ABZI_D11 | 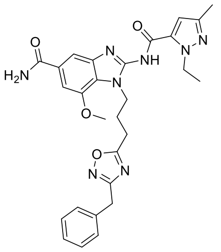 | 5.51 | 5.38 |
| 30 | ABZI_D16 | 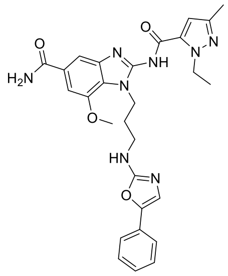 | 6.75 | 6.28 |
| 31 | ABZI_D17 | 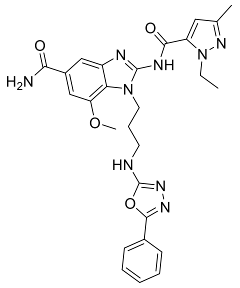 | 5.80 | 6.00 |
| 32 | ABZI_D18 | 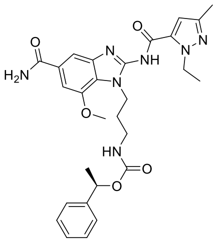 | 6.44 | 6.29 |
| 33 | ABZI_D19 | 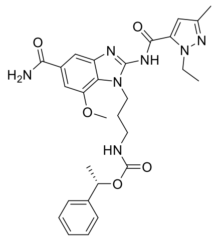 | 6.34 | 6.29 |

| | | | | |
|---|---|---|---|---|
| 34 | ABZI_D20 | 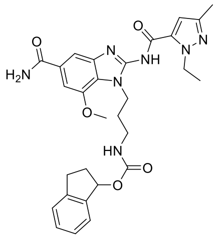 | 6.62 | 6.23 |
| 35 | ABZI_D21 | 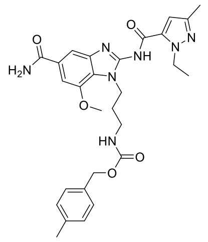 | 6.53 | 6.33 |
| 36 | ABZI_D22 | 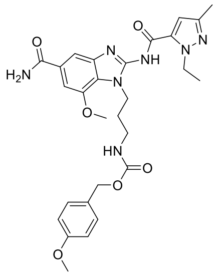 | 6.27 | 6.34 |
| 37 | ABZI_D23 | 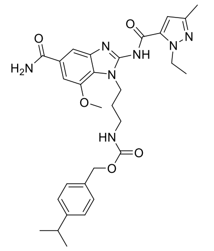 | 5.79 | 6.35 |
| 38 | ABZI_D24 | 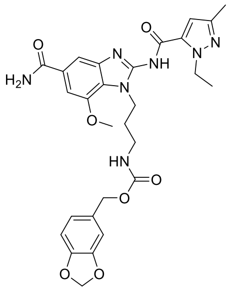 | 6.49 | 6.39 |
| 39 | ABZI_D25 | 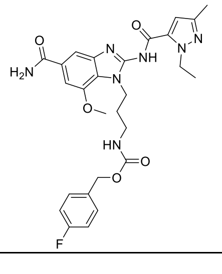 | 6.72 | 6.32 |

| | | | | |
|---|---|---|---|---|
| 40 | ABZI_D26 | 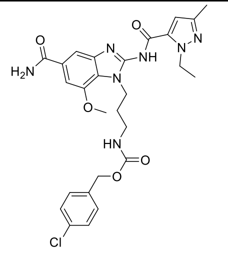 | 6.49 | 6.33 |
| 41 | ABZI_D27 | 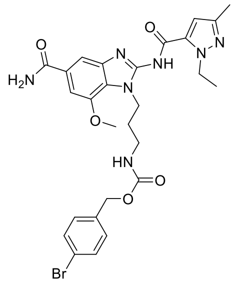 | 6.28 | 6.33 |
| 42 | ABZI_D28 | 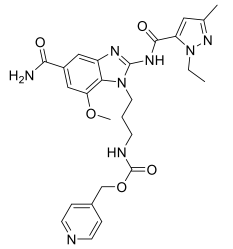 | 5.54 | 6.29 |
| 43 | ABZI_D29 | 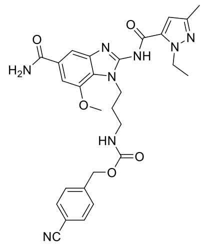 | 6.15 | 6.22 |
| 44 | ABZI_D30 | 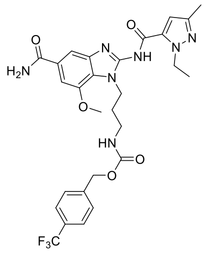 | 5.96 | 6.23 |
| 45 | ABZI_D32 | 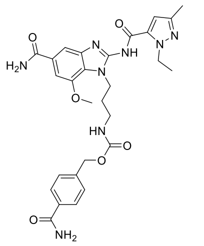 | 6.18 | 6.29 |
| 46 | ABZI_D33 | 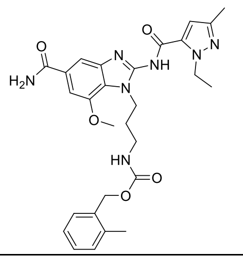 | 6.17 | 6.34 |

| | | | | |
|---|---|---|---|---|
| 47 | ABZI_D34 | 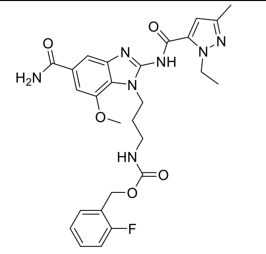 | 6.65 | 6.45 |
| 48 | ABZI_D35 | 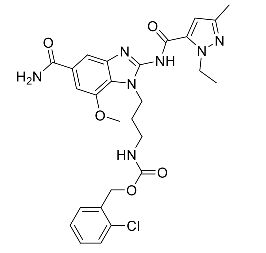 | 6.93 | 6.44 |
| 49 | ABZI_D36 | 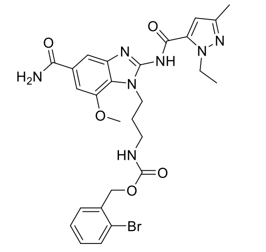 | 6.90 | 6.44 |
| 50 | ABZI_D37 | 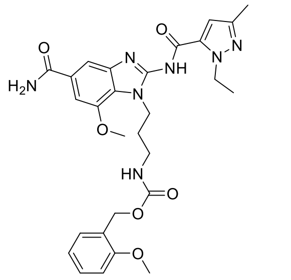 | 6.72 | 6.32 |
| 51 | ABZI_D38 | 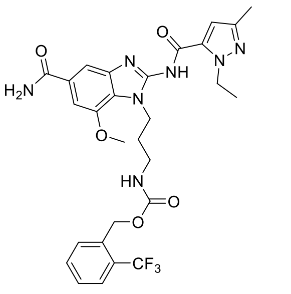 | 6.58 | 6.57 |
| 52 | ABZI_D39 | 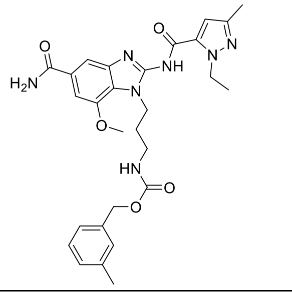 | 6.92 | 6.33 |

| | | | | |
|---|---|---|---|---|
| 53 | ABZI_D4 | 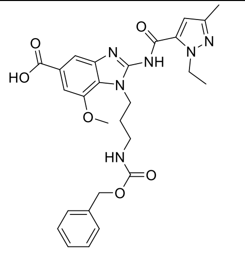 | 4.70 | 5.58 |
| 54 | ABZI_D40 | 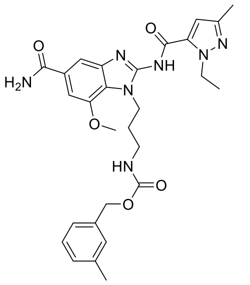 | 6.49 | 6.38 |
| 55 | ABZI_D41 | 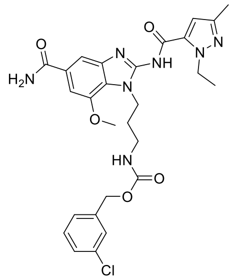 | 6.23 | 6.38 |
| 56 | ABZI_D42 | 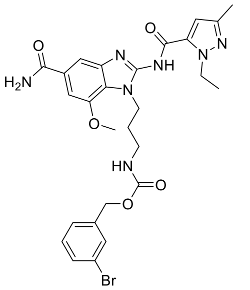 | 6.60 | 6.38 |
| 57 | ABZI_D43 | 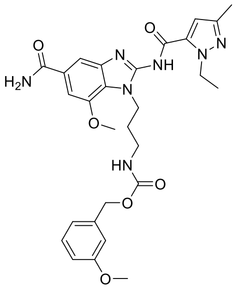 | 6.51 | 6.35 |
| 58 | ABZI_D44 | 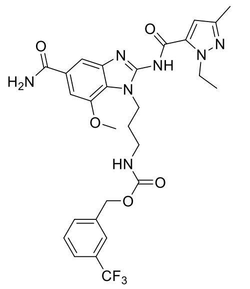 | 6.15 | 6.35 |

| | | | | |
|---|---|---|---|---|
| 59 | ABZI_D45 | 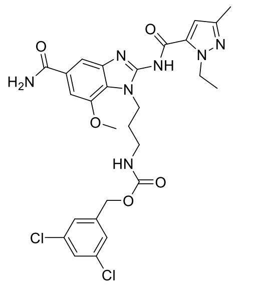 | 6.05 | 6.33 |
| 60 | ABZI_D46 | 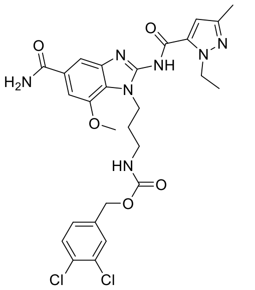 | 6.16 | 6.38 |
| 61 | ABZI_D47 | 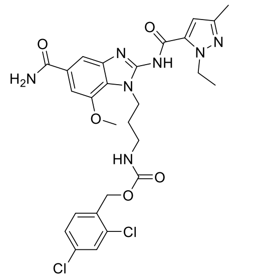 | 6.41 | 6.44 |
| 62 | ABZI_D48 | 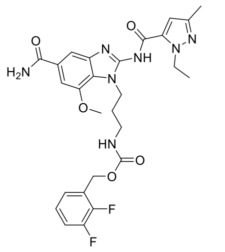 | 6.99 | 6.50 |
| 63 | ABZI_D49 | 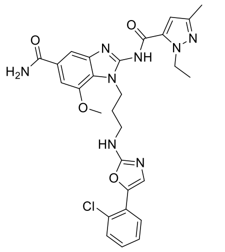 | 6.59 | 6.44 |
| 64 | ABZI_D5 | 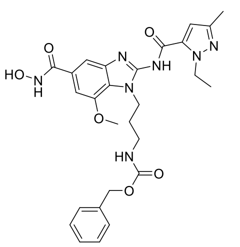 | 5.98 | 6.03 |

| | | | | |
|---|---|---|---|---|
| 65 | ABZI_D50 | 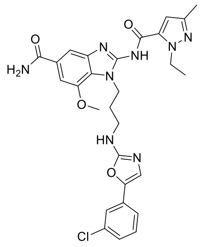 | 6.30 | 6.27 |
| 66 | ABZI_D51 | 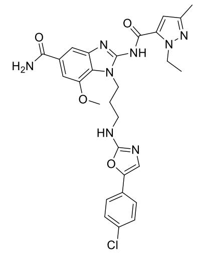 | 6.19 | 6.25 |
| 67 | ABZI_D52 | 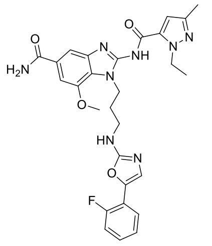 | 6.45 | 6.47 |
| 68 | ABZI_D53 | 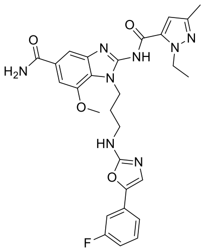 | 6.62 | 6.27 |
| 69 | ABZI_D54 | 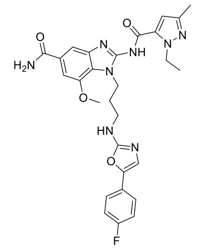 | 6.42 | 6.24 |
| 70 | ABZI_D55 | 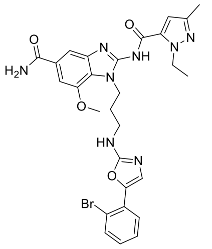 | 7.00 | 6.46 |
| 71 | ABZI_D56 | 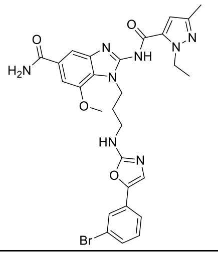 | 6.14 | 6.28 |

| | | | | |
|---|---|---|---|---|
| 72 | ABZI_D57 | 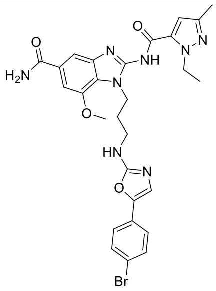 | 5.95 | 6.24 |
| 73 | ABZI_D58 | 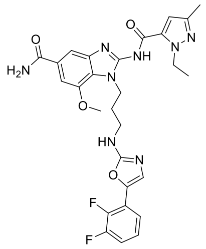 | 6.71 | 6.45 |
| 74 | ABZI_D59 | 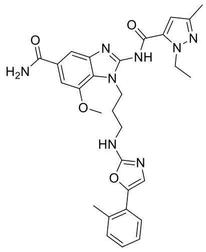 | 7.19 | 6.34 |
| 75 | ABZI_D6 | 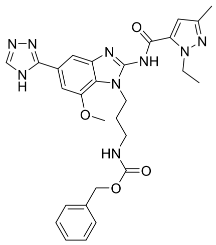 | 4.93 | 4.43 |
| 76 | ABZI_D60 | 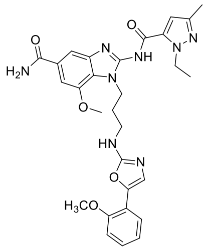 | 6.13 | 6.50 |
| 77 | ABZI_D61 | 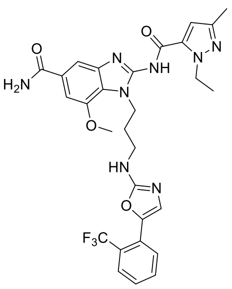 | 7.08 | 6.63 |

| | | | | |
|---|---|---|---|---|
| 78 | ABZI_D62 | 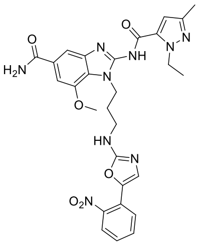 | 6.34 | 6.58 |
| 79 | ABZI_D63 | 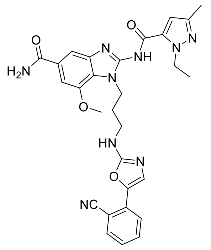 | 6.33 | 6.61 |
| 80 | ABZI_D64 | 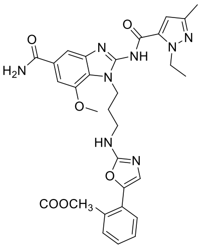 | 6.43 | 6.53 |
| 81 | ABZI_D65 | 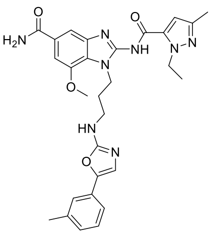 | 6.57 | 6.30 |
| 82 | ABZI_D66 | 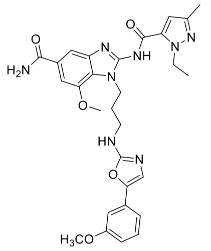 | 6.13 | 6.27 |
| 83 | ABZI_D67 | 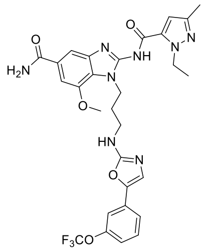 | 6.21 | 6.3 |
| 84 | ABZI_D69 | 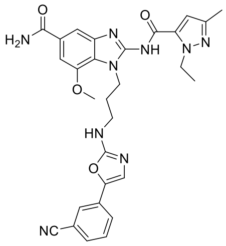 | 6.22 | 6.25 |

| | | | | |
|---|---|---|---|---|
| 85 | ABZI_D7 | 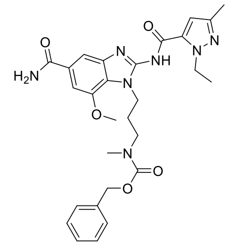 | 5.70 | 6.19 |
| 86 | ABZI_D71 | 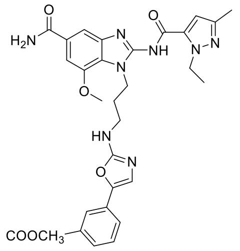 | 6.25 | 6.14 |
| 87 | ABZI_D73 | 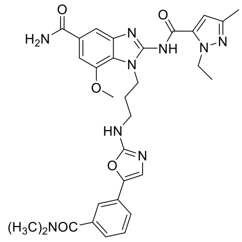 | 5.31 | 6.14 |
| 88 | ABZI_D8 | 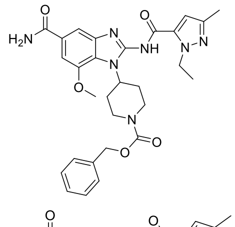 | 6.05 | 5.59 |
| 89 | ABZI_D9 | 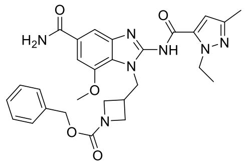 | 5.48 | 5.22 |
| 90 | ABZI_com51 | 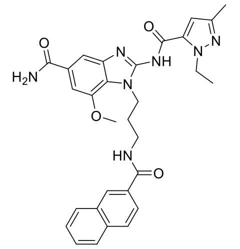 | 6.03 | 6.09 |
| 91 | ABZI_com57 | 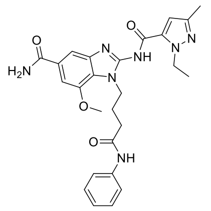 | 5.01 | 5.66 |
| 92 | ABZI_com58 | 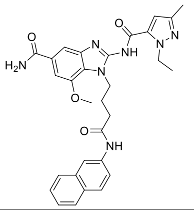 | 5.01 | 5.83 |

| | | | | |
|---|---|---|---|---|
| 93 | ABZI_com59 | 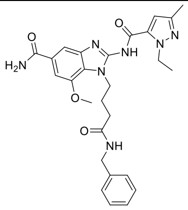 | 5.38 | 5.56 |
| 94 | ABZI_com60 | 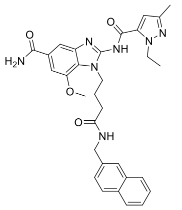 | 5.15 | 5.61 |
| 95 | ABZI_com69 | 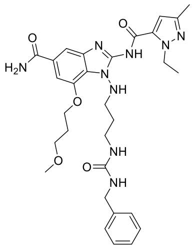 | 5.07 | 5.79 |
| 96 | ABZI_com72 | 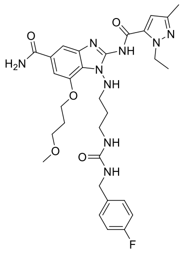 | 5.80 | 5.78 |
| 97 | ABZI_com73 | 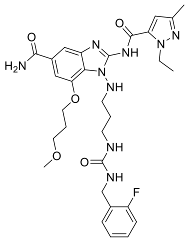 | 5.38 | 5.89 |
| 98 | ABZI_com77 | 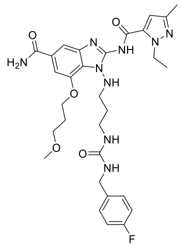 | 5.49 | 5.78 |
| 99 | ABZI_com88 | 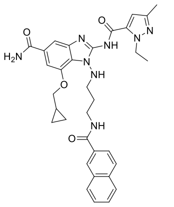 | 5.95 | 5.58 |

| | | | | |
|---|---|---|---|---|
| 100 | ABZI_com89 | 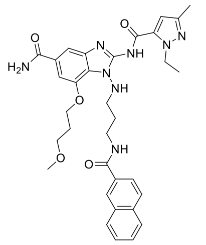 | 6.28 | 5.97 |
| 101 | ABZI_com90 | 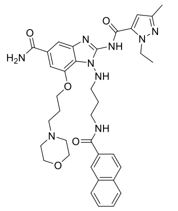 | 5.66 | 5.74 |
| 102 | ABZI_com91 | 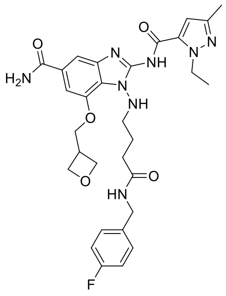 | 5.15 | 5.69 |
| 103 | ABZI_com93 | 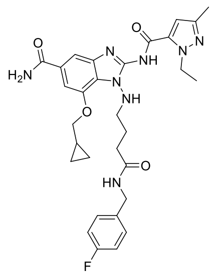 | 5.85 | 5.64 |
| 104 | ABZI_com94 | 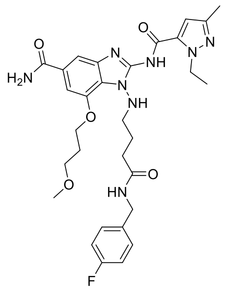 | 5.53 | 6.03 |
| 105 | ABZI_com95 | 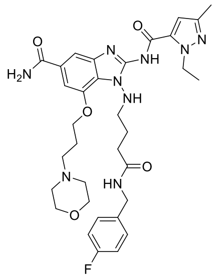 | 5.01 | 5.80 |

| | | | | |
|---|---|---|---|---|
| 106 | ABZI_SAP03 | 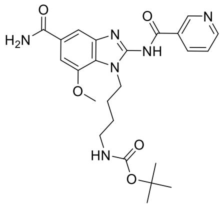 | 4.38 | 5.20 |
| 107 | ABZI_SAP04 | 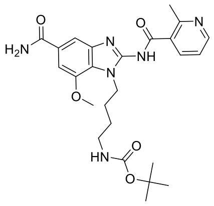 | 5.51 | 5.45 |
| 108 | ABZI_SAP16 | 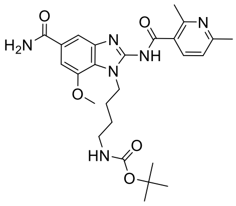 | 4.53 | 5.48 |
| 109 | ABZI_SAP2a | 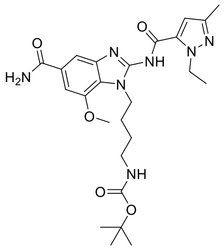 | 4.71 | 5.66 |

**Table S2.** Compound molecular docking score top50.

| Compounds name | Scores | London dG | Compounds name | Scores | London dG |
|---|---|---|---|---|---|
| M1 | -9.83 | -14.96 | M26 | -9.44 | -15.15 |
| M2 | -9.78 | -15.25 | M27 | -9.43 | -15.20 |
| M3 | -9.68 | -15.18 | M28 | -9.43 | -15.07 |
| M4 | -9.67 | -15.35 | M29 | -9.41 | -15.20 |
| M5 | -9.66 | -14.80 | M30 | -9.41 | -15.74 |
| M6 | -9.65 | -15.35 | M31 | -9.41 | -15.98 |
| M7 | -9.63 | -16.36 | M32 | -9.41 | -15.25 |
| M8 | -9.61 | -15.77 | M33 | -9.39 | -15.65 |
| M9 | -9.61 | -15.31 | M34 | -9.38 | -15.03 |
| M10 | -9.58 | -15.28 | M35 | -9.38 | -15.71 |
| M11 | -9.58 | -16.41 | M36 | -9.37 | -15.08 |
| M12 | -9.57 | -15.16 | M37 | -9.37 | -15.19 |
| M13 | -9.57 | -15.15 | M38 | -9.37 | -15.19 |
| M14 | -9.57 | -15.83 | M39 | -9.37 | -15.08 |
| M15 | -9.57 | -15.97 | M40 | -9.37 | -14.96 |
| M16 | -9.56 | -15.73 | M41 | -9.37 | -15.97 |
| M17 | -9.56 | -15.27 | M42 | -9.36 | -15.26 |

| | | | | | |
|---|---|---|---|---|---|
| M18 | -9.55 | -15.06 | M43 | -9.36 | -16.09 |
| M19 | -9.52 | -15.24 | M44 | -9.36 | -15.16 |
| M20 | -9.50 | -15.75 | M45 | -9.36 | -15.71 |
| M21 | -9.49 | -15.10 | M46 | -9.35 | -15.85 |
| M22 | -9.48 | -15.10 | M47 | -9.35 | -15.15 |
| M23 | -9.45 | -15.88 | M48 | -9.34 | -15.66 |
| M24 | -9.45 | -15.82 | M49 | -9.34 | -15.75 |
| M25 | -9.45 | -15.14 | M50 | -9.34 | -15.60 |

**Table S3.** The prediction results of DeepSA. HA_num rule_of_five represent the number of heavy atoms, Lipinski's Rule of Five respectively. ES and HS represent probability values, a molecule requires less than or equal to 10 synthetic steps was labeled as ES, otherwise, if the required step is larger than 10 or can't be successfully predicted by Retro* was labeled as HS.

| Id | easy-to-synthesize (ES) | hard-to-synthesize (HS) groups | HA_num | RingSystem_num | Ring_num | rule_of_five |
|---|---|---|---|---|---|---|
| D59 | 0.9971 | 0.0029 | 41 | 4 | 5 | 0 |
| M11 | 0.9707 | 0.0293 | 50 | 4 | 5 | 0 |
| M13 | 0.7776 | 0.2224 | 49 | 4 | 5 | 0 |
| M44 | 0.9588 | 0.0412 | 49 | 4 | 6 | 0 |

**Table S4.** The prediction results of RAscore using XGB model . The values represent synthetic accessibility probability.

| Id | RAscore |
|---|---|
| D59 | 0.81 |
| M11 | 0.33 |
| M13 | 0.59 |
| M44 | 0.78 |

We used models such as SAscore, DeepSA and RAscore to make predictions, further illustrating the synthetic accessibility of the compounds [1]. As shown in **Table 6**, the SAscore of these compounds indicate that they are all relatively easy to synthesize. As shown Table S3, The ES probabilities are all between 0.77 and 0.997, and the HS probabilities are only between 0.0029 and 0.222, far below the threshold (0.47/0.5). The model indicates that these compounds

are likely easy to synthesize, with low synthesis difficulty. Furthermore, these compounds are all 4-, 5-, or 6-ring systems with moderate ring structure complexity, without excessive fused rings/complex ring systems (such structures increase synthesis difficulty). These six compounds' rule_of_five is 0 indicates that it fully meets the characteristics of a drug-like substance, has no obvious structural defects (such as excessive diversity of atoms, abnormal hydrogen bond donors/acceptors), and has high application potential after synthesis. In addition, as shown Table S4, it showed good synthetic accessibility with the compounds except for M11.

Overall, these five hit compounds exhibited good synthetic accessibility, indicating that they could be synthesized experimentally.

**Figure S1.** Predicted synthetic route of compound M13.

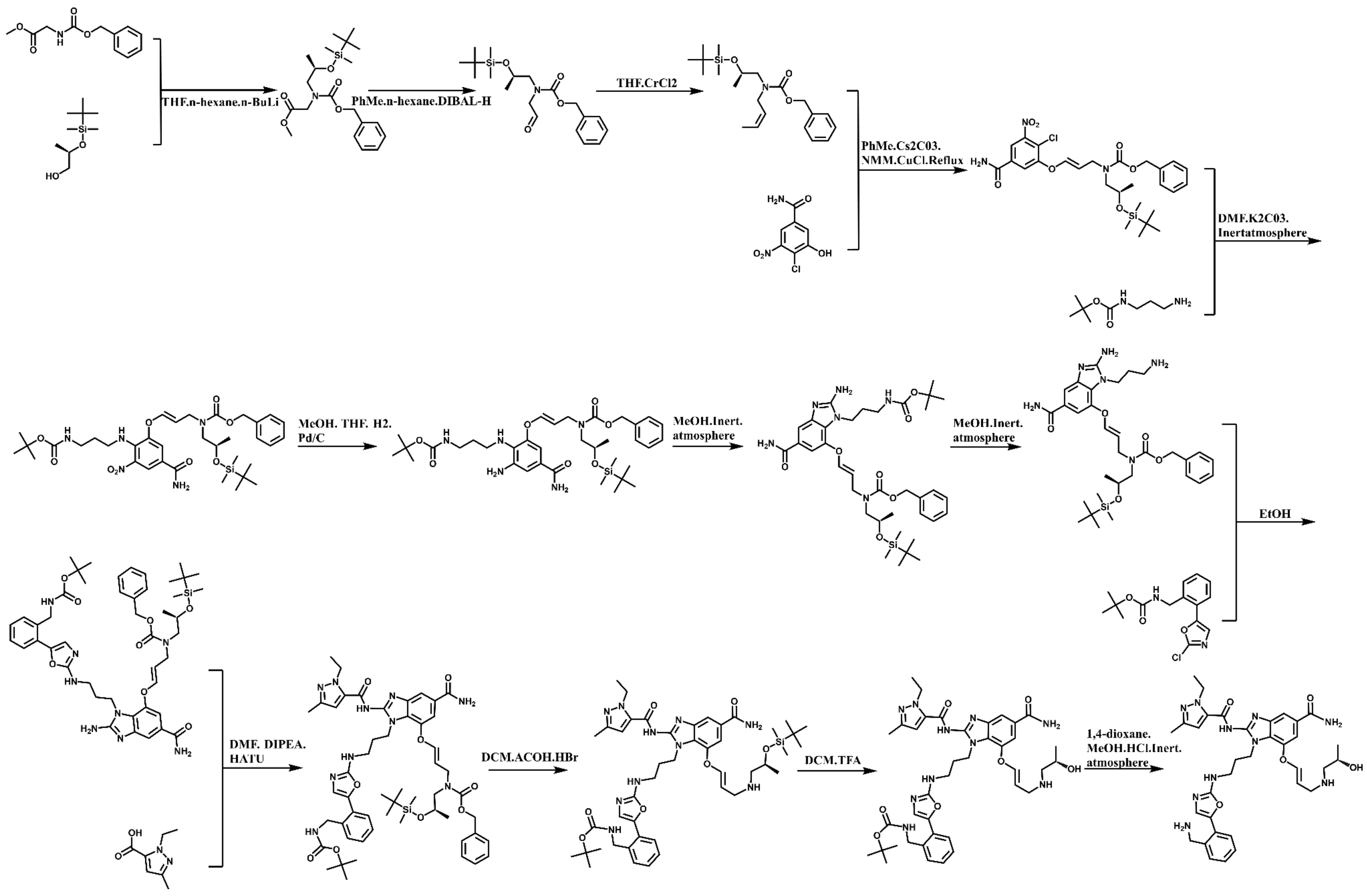
THF.n-hexane.n-BuLi
PhMe.n-hexane.DIBAL-H
THF.CrCl2
PhMe.Cs2C03.
NMM.CuCl.Reflux
DMF.K2C03.
Inertatmosphere
MeOH. THF. H2.
Pd/C
MeOH.Inert.
atmosphere
MeOH.Inert.
atmosphere
EtOH
DMF. DIPEA.
HATU
DCM.ACOH.HBr
DCM.TFA
1,4-dioxane.
MeOH.HCl.Inert.
atmosphere

**Figure S2.** Details of thepredicted synthetic route for compound M13.

| | |
|---|---|
| **Export Time** | 2025-08-21 20:41:50 |
| **Export Route** | R005 |
| **Target Molecule** | 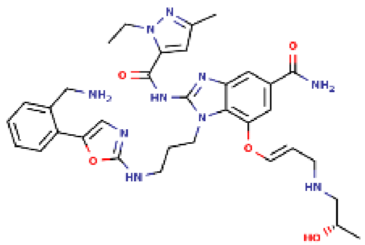 |
| **Comment** | |

| | |
|---|---|
| **Export Route** | 3a1bde40-ad11-92aa-ad18-95e0616b7745_R005 |
| **Scheme** | |

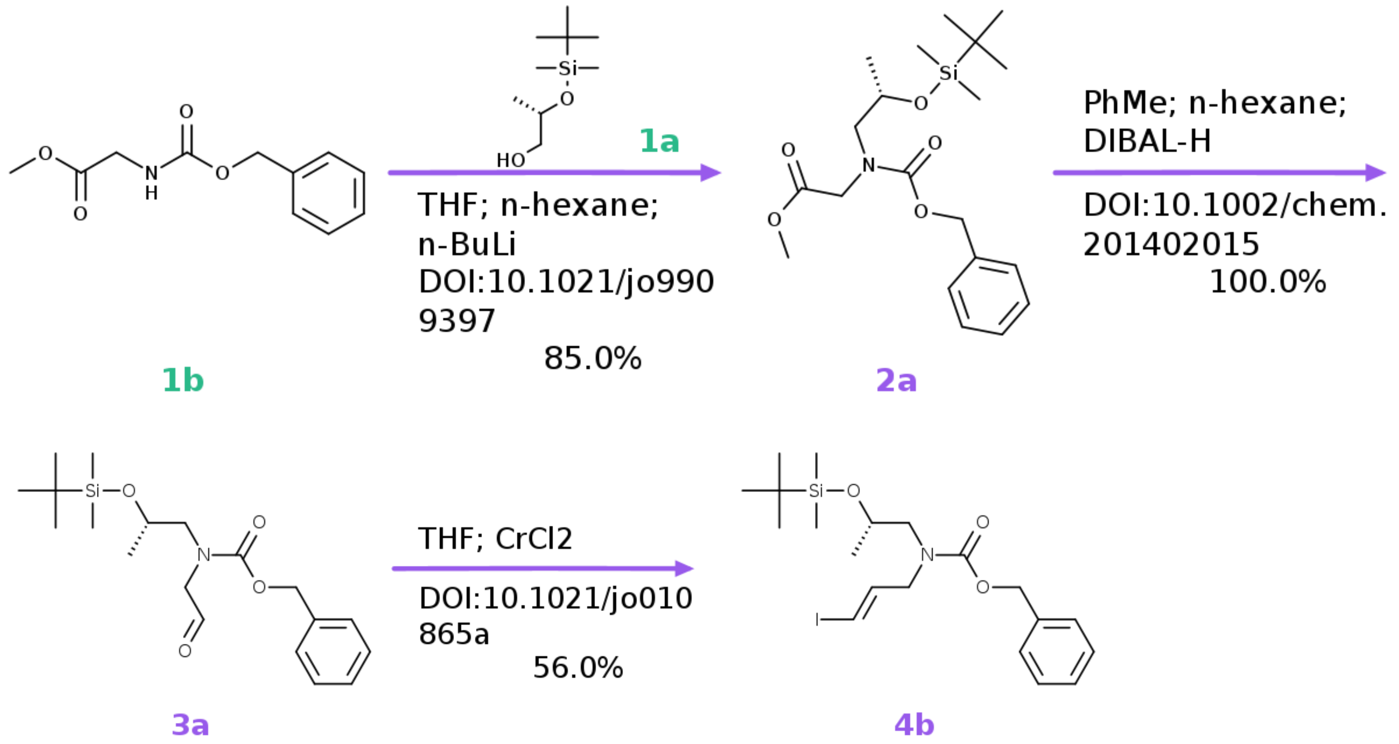
1a
THF; n-hexane;
n-BuLi
DOI:10.1021/jo990
9397
85.0%
1b
2a
PhMe; n-hexane;
DIBAL-H
DOI:10.1002/chem.
201402015
100.0%
THF; CrCl2
DOI:10.1021/jo010
865a
56.0%
3a
4b

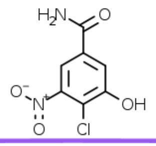

4a

PhMe; Cs2CO3; NMM; CuCl; Reflux
DOI:10.1002/anie.201204835
45.0%

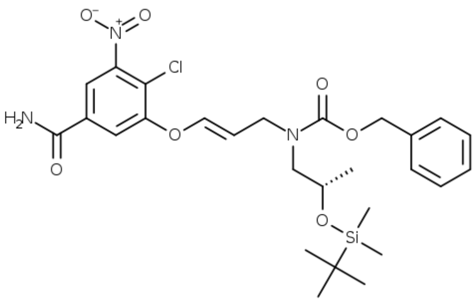

5b

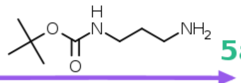

5a

DMF; K2CO3; Inert atmosphere
56.28%

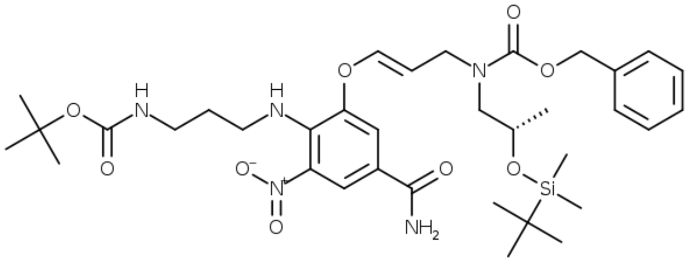

6a

MeOH; THF; H2; Pd/C

DOI:10.1021/acs.jmedchem.2c02046

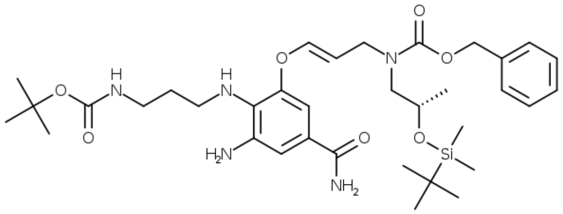

7a

MeOH; Inert atmosphere
52.7%

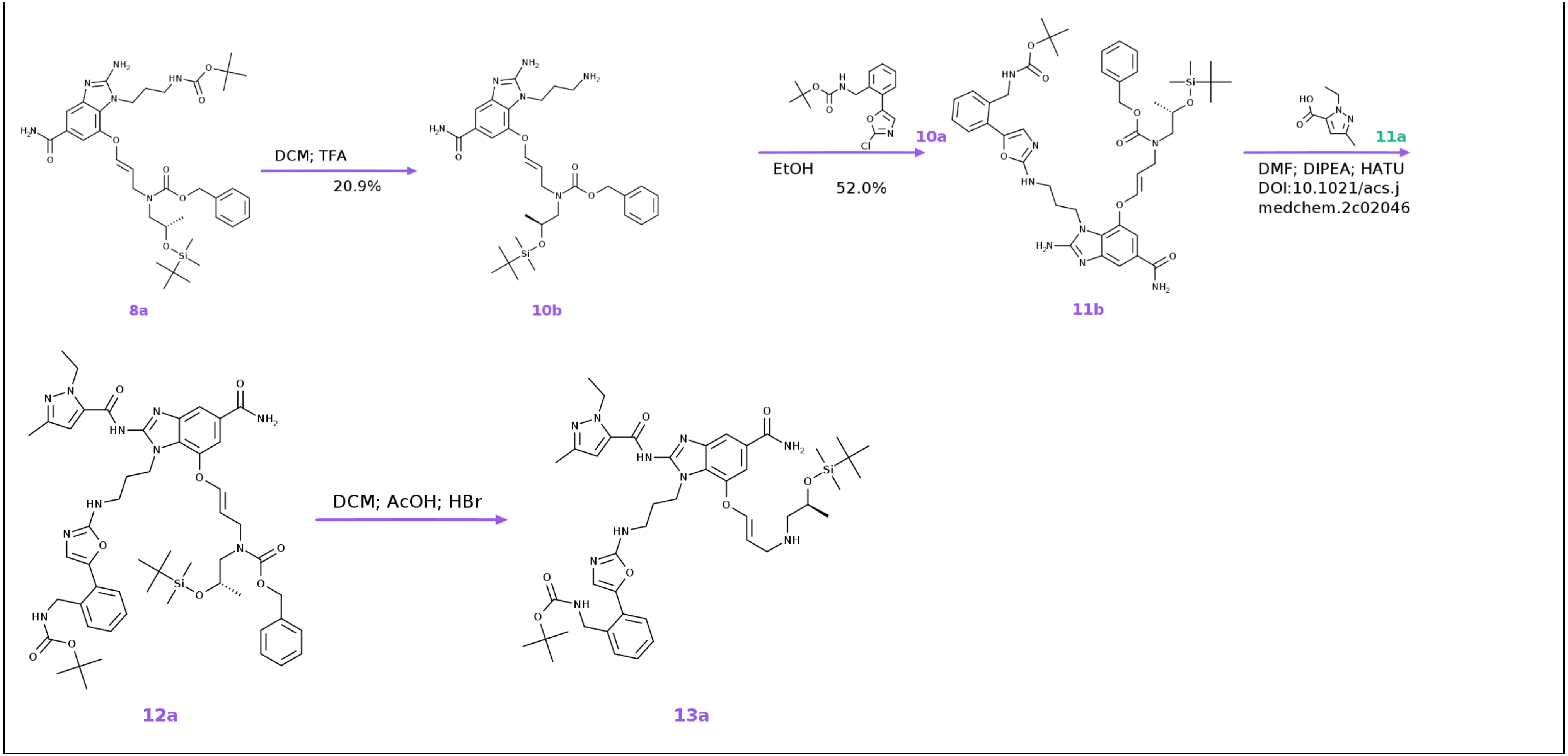

8a
DCM; TFA
20.9%
10b
10a
EtOH
52.0%
11b
11a
DMF; DIPEA; HATU
DOI:10.1021/acs.j
medchem.2c02046
12a
DCM; AcOH; HBr
13a

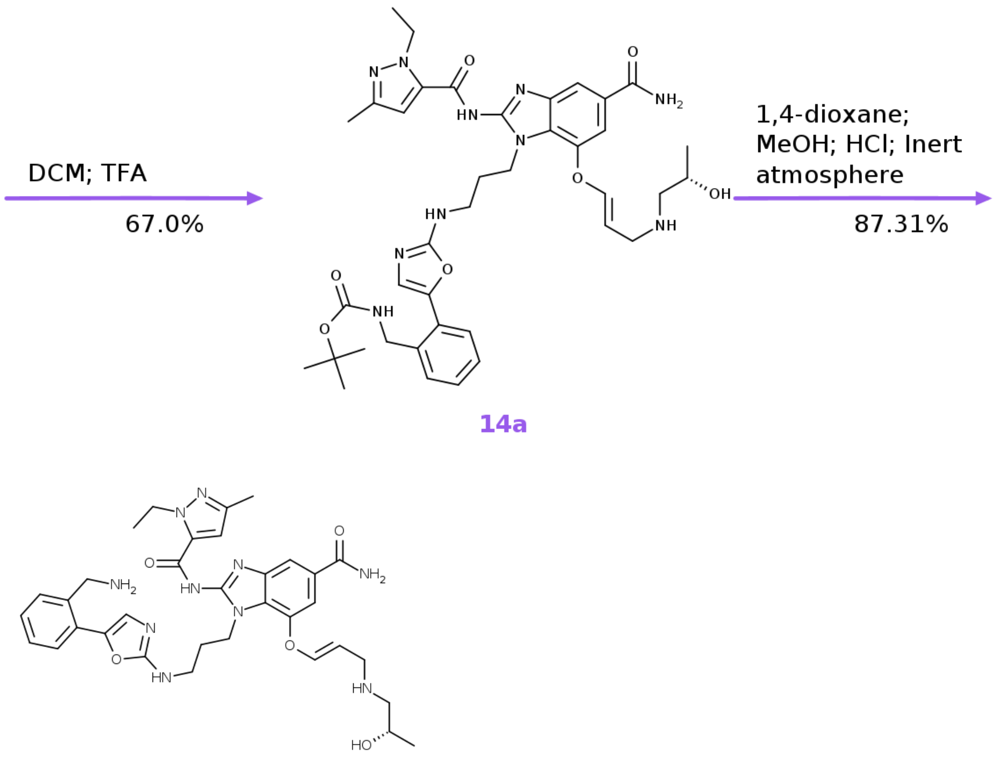


**14a**

**Target Compound**

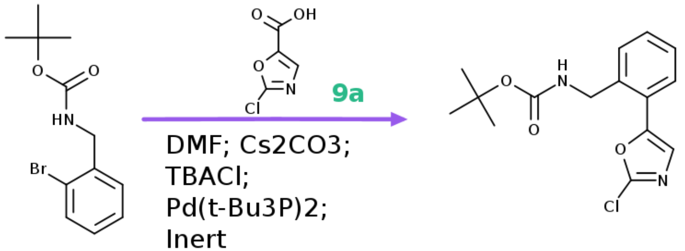

9a

9b

DMF; Cs2CO3; TBACl; Pd(t-Bu3P)2; Inert atmosphere; Microwave

52.0%

10a

Information_3a1bde40-ad11-92aa-ad18-95e0616b7745_R005 View in ChemAIRS

| ID | Target Compound (condition 1) |
| --- | --- |
| ReferenceRXN | 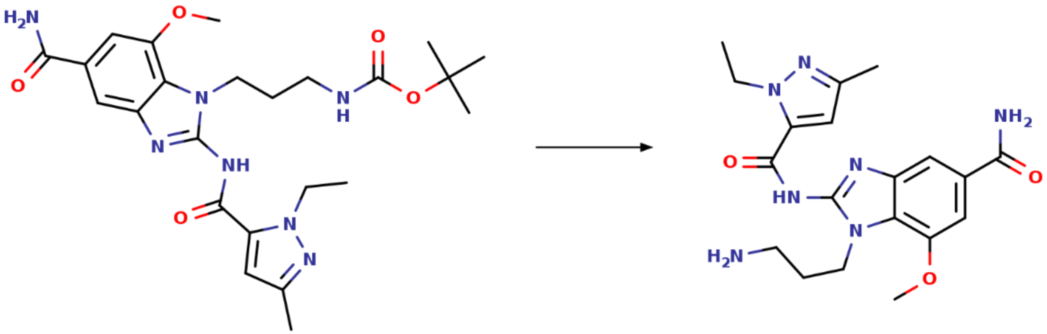 |

| | |
|---|---|
| **CAS** | N/A;N/A |
| **Yield(%)** | 88 |
| **Similarity(%)** | 81 |
| **Conditions** | 1,4-dioxane; MeOH; HCl; Inert atmosphere |
| **Procedures** | Into a 250-mL 3-necked round-bottom flask purged and maintained with an inert atmosphere of nitrogen, was placed 20.4 (1.59 g, 3.18 mmol, 1 eq) in MeOH (40 mL) and dioxane (30 mL). The resulting solution was stirred for 12 h at 25Â° C. The resulting mixture was concentrated under vacuum. This resulted in 1.11 g (87.31 percent) of 20.5 as a red solid. (ES, m/z): 400 (M+H)+. |
| **References** | WO2020/132566; A1; (2020) [2] |
| **DOI/Link** | N/A |
| **ID** | Target Compound (condition 2) |

| | |
|---|---|
| **ReferenceRXN** | 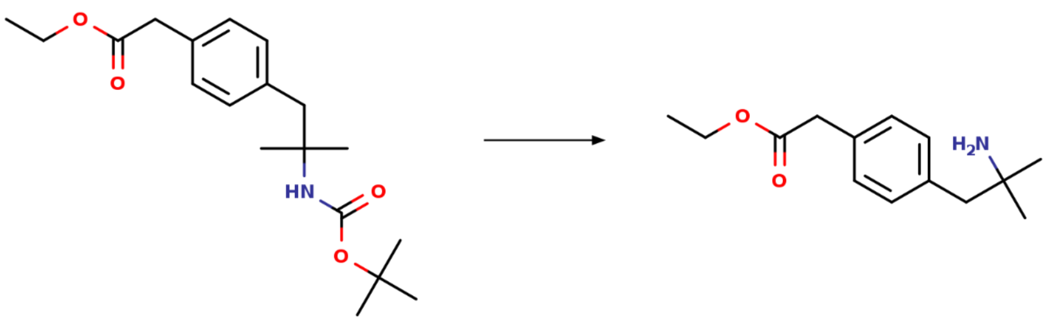 |
| **CAS** | 1251957-73-9;808768-89-0 |
| **Yield(%)** | 100 |
| **Similarity(%)** | N/A |
| **Conditions** | H2O; HCl; K2CO3; 1,4-dioxane; EtOH |
| **Procedures** | # Synthesis of the Ethyl [4-(2-Amino-2-methylpropyl)phenyl]acetate□□HCl (4 M) in dioxane (30 mL) was added to a stirred solution of the ester 5 (3.75 g, 11.18 mmol) in ethanol (30 mL) and the mixture stirred at room temperature overnight. The reaction mixture was evaporated in Vacuo and 10% aqueous potassium carbonate added until the pH of the mixture reached 8. The mixture was extracted with ethyl acetate (3 150 mL), and the combined extracts were washed with brine, dried over MgSO4, and evaporated in Vacuo to give the title compound 6 as a yellow oil (2.72 g, 100%). 1H NMR (400 MHz, CDCl3) δ: 1.10 (s, 6H), 1.24 (t, J ) 7.2 Hz, 3H) 2.64 (s, 2H), 3.58 (s, 2H), 4.16 (q, J ) 7.2 Hz, 2H), 7.15 7 (br d, J ) 8.2 Hz, 2H), 7.22 (br d, J ) 8.2 Hz, 2H); LC/MS Rt ) 0.73 min, m/z |

| | |
|---|---|
| | (ES+) 236 [MH+]. |
| **References** | Organic Process Research and Development; vol. 14; 6; (2010); p. 1326 - 1336 [3] |
| **DOI/Link** | 10.1021/op1001462 |
| **ID** | Target Compound (condition 3) |
| **ReferenceRXN** | 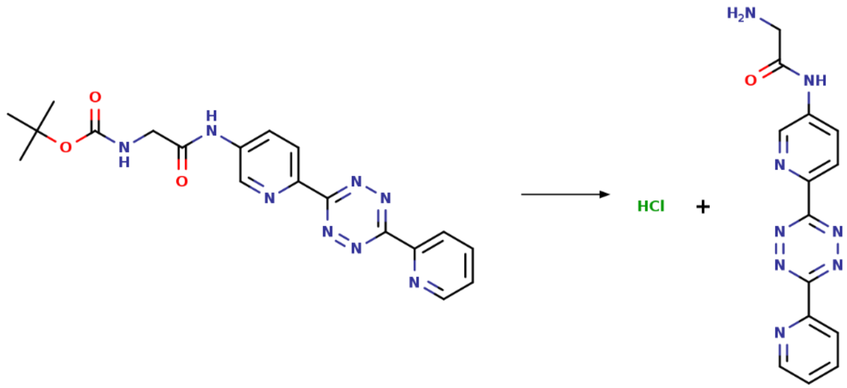 |
| **CAS** | 1360467-31-7;7647-01-0;1360535-79-0 |
| **Yield(%)** | 100 |
| **Similarity(%)** | 27 |

| | |
|---|---|
| **Conditions** | HCl; 1,4-dioxane; DCM |
| **Procedures** | Boc-protected Tetrazine S6 was synthesized using the procedure reported earlier6. 4M HCl in dioxane (500 mu,, 2,0 mmol) was added to a stirring solution of Tetrazine S5 (8 mg, 0.02 mmol) in DCM (500 muL). The reaction was carried out for 2 h at room temperature and subsequently the solvent was removed under reduced pressure to yield primary amine hydrochloride S6 as a pink solid (6mg, 0.02 mmol, 100 percent). The compound was directly used in the next step without any further purification. |
| **References** | WO2015/136265; A1; (2015) [4] |
| **DOI/Link** | N/A |
| **ID** | 14a (condition 1) |
| **ReferenceRXN** | 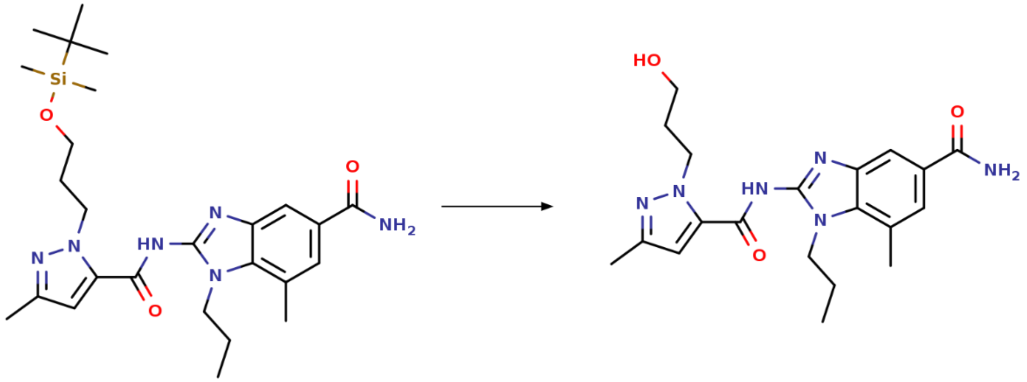 |
| **CAS** | N/A;N/A |

| | |
|---|---|
| **Yield(%)** | 67 |
| **Similarity(%)** | 82 |
| **Conditions** | DCM; TFA |
| **Procedures** | To a solution of 51.3 (120 mg, 0.234 mmol, 1 equiv) in dichloromethane (10 mL) was added trifluoroacetic acid (3 mL). The resulting mixture was stirred at RT for Ih. The mixture was concentrated under reduced pressure. The crude product was purified by Prep-HPLC with the following conditions: Column, X select CSH OBD Column 30* 150mm, 5um; mobile phase, water (0.1 percent FA) and ACN (47 percent ACN up to 57 percent in 7 min); UV detection at 254/220 nm. This resulted in 62.5 mg (67 percent) of 1-127 as an off-white solid. (ES, m/z): 399 (M+H+); 1H NMR (400 MHz, DMSO-d6) d 12.83 (s, 1H), 7.90-7.87 (m, 2H), 7.58 (s, 1H), 7.30 (s, 1H), 6.65 (s, 1H), 4.66 (t, 2H), 4.53 (t, 1H), 4.33 (t, 2H), 3.46-3.41 (m, 2H), 2.67 (s, 3H), 2.18 (s, 3H), 1.96-1.91 (m, 2H), 1.80-1.74 (m, 2H), 0.97 (t, 3H). |
| **References** | WO2020/132566; A1; (2020) [2] |
| **DOI/Link** | N/A |
| **ID** | 14a (condition 2) |

| | |
|---|---|
| **ReferenceRXN** | 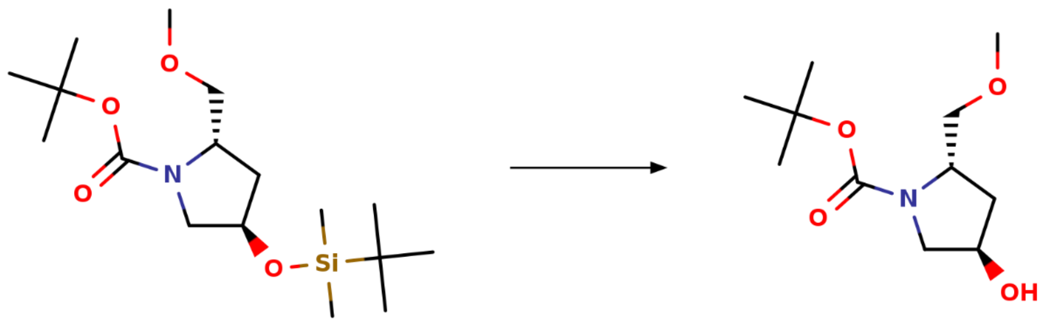 |
| **CAS** | N/A;132945-85-8 |
| **Yield(%)** | 100 |
| **Similarity(%)** | 24 |
| **Conditions** | TBAF; THF |
| **Procedures** | 4E (6.2 g, 17.94 mmol) was dissolved in tetrahydrofuran (60 mL) at room temperature, Tetrabutylammonium fluoride (9.38 g, 35.88 mmol) was added and reacted at room temperature for 2 hours. Water (60 mL) was added to the reaction solution, and the mixture was extracted with ethyl acetate (50 mL x 4). The organic phases were combined and washed with saturated brine solution (40 mL x 1). Dried over anhydrous magnesium sulfate, filtered, and the filtrate was concentrated under reduced pressure to give a pale yellow liquid 4F (5.0 g, yield 100 percent). |

| ID | 14a (condition 3) |
| --- | --- |
| ReferenceRXN | 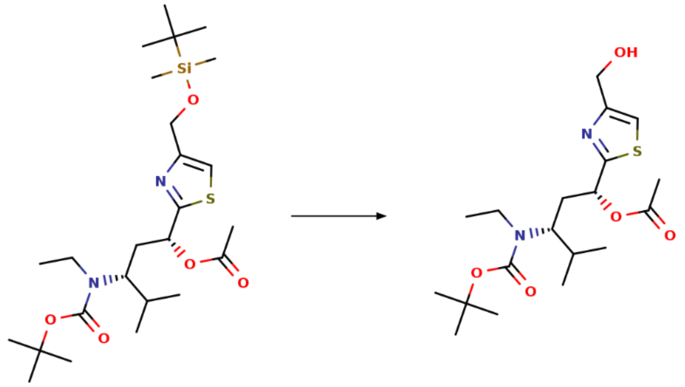 |
| CAS | N/A;N/A |
| Yield(%) | 100 |
| Similarity(%) | 36 |
| Conditions | TBAF; THF |
| Procedures | N/A |
| References | Organic Process Research and Development; vol. 21; 10; (2017); p. 1602 - 1609 [5] |

| | |
|---|---|
| **DOI/Link** | 10.1021/acs.oprd.7b00232 |
| **ID** | 13a (condition 1) |
| **ReferenceRXN** | 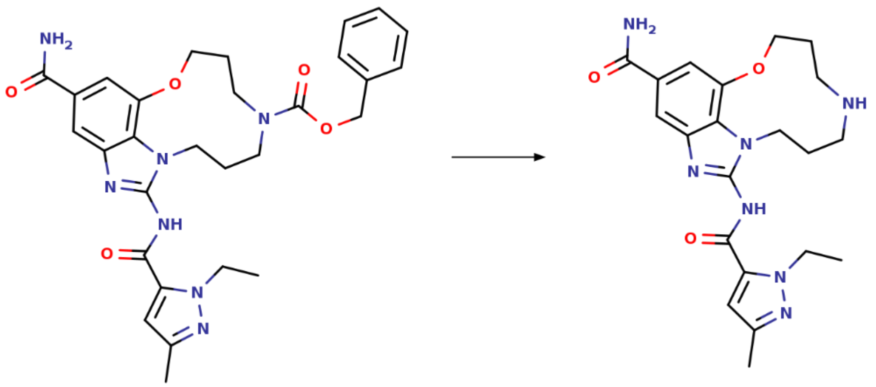 |
| **CAS** | N/A;N/A |
| **Yield(%)** | N/A |
| **Similarity(%)** | 76 |
| **Conditions** | DCM; AcOH; HBr |
| **Procedures** | Disperse 21i (130 mg, 0.23 mmol) in dichloromethane (5 mL), add hydrobromic acid/acetic acid (33%, 5 mL), and react at room temperature for 1 h. The solvent was spin-dried to obtain the crude product (140 mg, hydrobromide and containing acetic acid), 50 |

| | |
|---|---|
| | mg of the crude product was separated and purified by preparative HPLC to obtain compound 21 (22 mg). |
| **References** | CN111471056; A; (2020) [6] |
| **DOI/Link** | N/A |
| **ID** | 13a (condition 2) |
| **ReferenceRXN** | 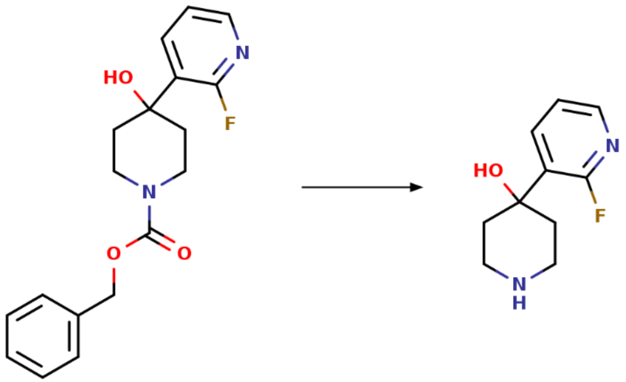 |
| **CAS** | 161610-13-5;360766-60-5 |
| **Yield(%)** | 100 |
| **Similarity(%)** | 27 |

| | |
|---|---|
| **Conditions** | Pd/C; H2; MeOH |
| **Procedures** | N/A |
| **References** | Organic Process Research and Development; vol. 5; 5; (2001); p. 491 - 497 [7] |
| **DOI/Link** | [10.1021/op0000935](https://doi.org/10.1021/op0000935) |
| **ID** | 13a (condition 3) |
| **ReferenceRXN** | 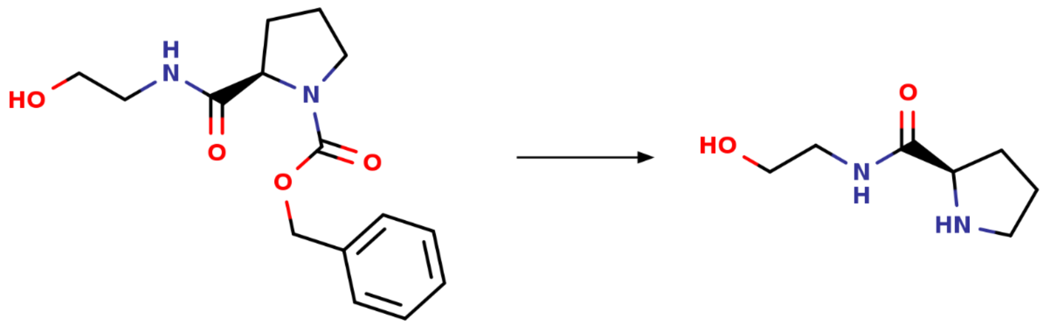 |
| **CAS** | N/A;N/A |
| **Yield(%)** | 100 |

| | |
|---|---|
| **Similarity(%)** | N/A |
| **Conditions** | Pd/C; H2; MeOH |
| **Procedures** | An oven-dried flask was charged with Cbz-D-proline or Cbz-L-proline (1.00 eq.) or a proline derivative and dry dichloromethane (0.20 mol/L). The solution was cooled to 0 C and triethylamine (1 .00 eq.) and isobutyl chloroformate (1.00 eq.) were added. The mixture was stirred for 0.5 h, and the relevant amine (1 .00 eq.) was added. The mixture was warmed to room temperature and stirred until complete conversion (mon- itored by TLC). The mixture was washed with aq. sat. NH4Cl, aq. sat. NaHCO3 and brine. Each aqueous layer was re-extracted with dichloromethane. The combined or- ganic layers were dried over Na2SO4, filtered and concentrated in vacuo. The crude intermediate could be purified or used in the following step without further purification. The intermediate (1.00 eq.) was dissolved in MeOH (0.40 mol/L), the flask was flushed with argon three times and Pd/C (10.0 wt.%, 5.00 mol%) was added in one portion. The mixture was evacuated and flushed with hydrogen five times. The black suspension was stirred at room temperature under a hydrogen atmosphere until com- plete conversion (monitored by TLC). The reaction mixture was filtered over a plug of celite and rinsed with methanol. (0107) Example 1 - (R)-N-(2-hvdroxyethyl)pyrrolidine-2-carboxamide (ent-VIIb) (0108) According to the procedure above: Cbz-D-proline (2.49 g, 10.0 mmol, 1.00 eq.), tri ethylamine (1.41 mL, 10.0 mmol, 1 .00 eq.), isobutyl chloroformate (1.30 mL, 10.0 mmol, 1.00 eq.) and ethanolamine (1.21 mL, 10.0 mmol, 1.00 eq.) were reacted to form the intermediate (2.08 g). (0109) The intermediate (2.03 g, 6.94 mmol, 1 .00 eq.) and Pd/C (10.0 wt.%, 368 mg, 347 pmol, 5.00 mol%) yielded organocatalyst (ent-VIIb) as a colorless liquid (1.10 g, quant.). |
| **References** | WO2019/228874; A1; (2019) [8] |
| **DOI/Link** | N/A |

| ID | 12a (condition 1) |
|---|---|
| ReferenceRXN | 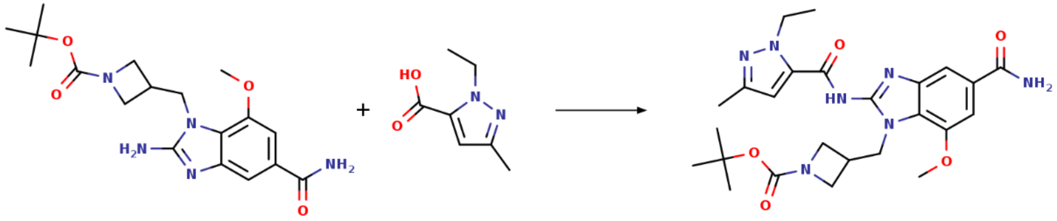 |
| CAS | N/A;50920-65-5;N/A |
| Yield(%) | N/A |
| Similarity(%) | 79 |
| Conditions | DMF; DIPEA; HATU |
| Procedures | N/A |
| References | Journal of Medicinal Chemistry; vol. 66; 8; (2023); p. 5584 - 5610 [9] |

| | |
|---|---|
| DOI/Link | 10.1021/acs.jmedchem.2c02046 |
| ID | 12a (condition 2) |
| ReferenceRXN | 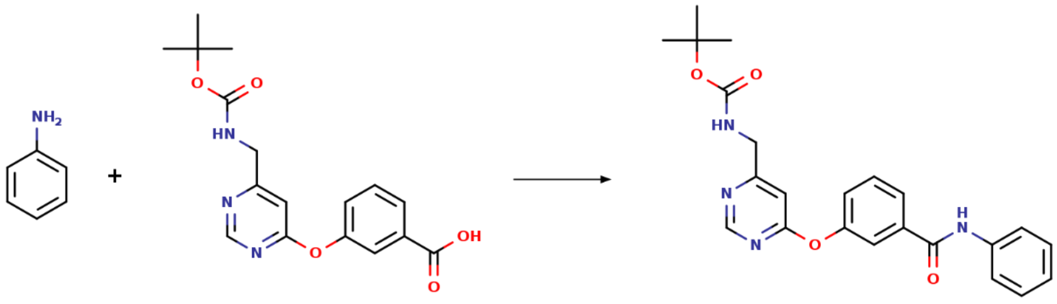 |
| CAS | 62-53-3;N/A;N/A |
| Yield(%) | 100 |
| Similarity(%) | 45 |
| Conditions | DIPEA; HATU; DCM |
| Procedures | To a stirred solution of mt-A (50 mg, 0.145 mmol) in DCM (2 mL), was added HATU (82 mg, 0.216 mmol) and the mixture was stirred at RT for 5 mm. Aniline (18 mg, 0.19 mmol) and DIEA (56 mg, 0.431 mmol)were added and the mixture stirred at RT for 16 h. The DCM was |

| | |
|---|---|
| | evaporated under reduced pressure and the remaining reaction mixture was partitioned between water (20 mL) and DCM (20 mL). The organic layer was separated, dried (Na2SO4), filtered, and then concentrated under reduced pressure. The crude residue was purified (silica gel; eluting with 0-100 percent EtOAc in hexanes), to afford compound 1 as a colorless oil (64 mg, 100 percent). ?H NMR (300 IVIFlz, CDC13): 7.87 (m, 1H), 7.62?7.72 (m, 4H), 7.51 (m, 1H), 7.32?7.39 (m, 4H), 7.15 (m, 1H), 7.02 (m, 1H), 5.08 (m, 1H), 4.37 ?4.40 (m, 2H), 1.46 (s, 9H); LCMS Mass:443.0 (M+Na). |
| **References** | WO2017/3862; A1; (2017) [10] |
| **DOI/Link** | N/A |
| **ID** | 12a (condition 3) |
| **ReferenceRXN** | 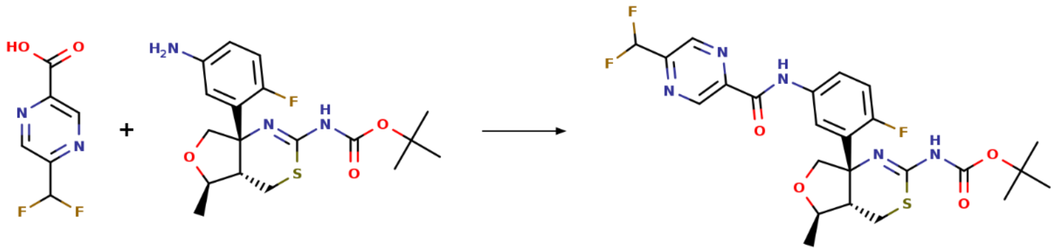 |
| **CAS** | 1174321-06-2;N/A;N/A |

| | |
|---|---|
| **Yield(%)** | 100 |
| **Similarity(%)** | 52 |
| **Conditions** | SOCl2; PhMe |
| **Procedures** | N/A |
| **References** | Organic Process Research and Development; (2021)[11] |
| **DOI/Link** | [10.1021/acs.oprd.1c00223](https://doi.org/10.1021/acs.oprd.1c00223) |
| **ID** | 11b (condition 1) |
| **ReferenceRXN** | 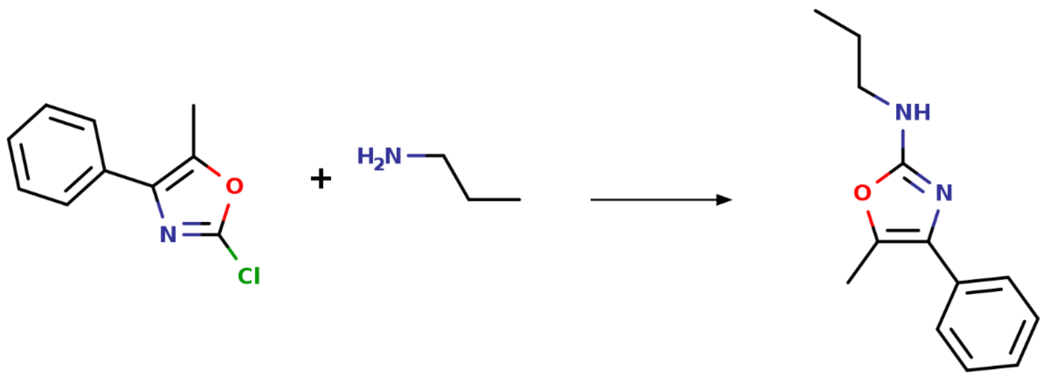 |

| | |
|---|---|
| **CAS** | 858114-76-8;107-10-8;858114-77-9 |
| **Yield(%)** | 52 |
| **Similarity(%)** | 35 |
| **Conditions** | EtOH |
| **Procedures** | Reference Example 22 5-methyl-4-phenyl-N-propyl-1,3-oxazole-2-amine; [Show Image] To a solution of 2-chloro-5-methyl-4-phenyl-1,3-oxazole (1.46 g, 7.56 mmol) in ethanol (15 mL) was added propylamine(5 mL), and the mixture was stirred in a sealed tube at 110° C for 8 hr. The reaction mixture was concentrated and water was added. The mixture was extracted with ethyl acetate, washed with saturated brine, dried over anhydrous magnesium sulfate, and concentrated under reduced pressure. The residue was purified by silica gel column chromatography (hexane/ethyl acetate=2:1) to give the title compound (850 mg, yield 52 percent) as a yellow powder. MS:m/z 217 (M+H). |
| **References** | EP1698624; A1; (2006) [12] |
| **DOI/Link** | N/A |
| **ID** | 11b (condition 2) |

| | |
|---|---|
| ReferenceRXN | 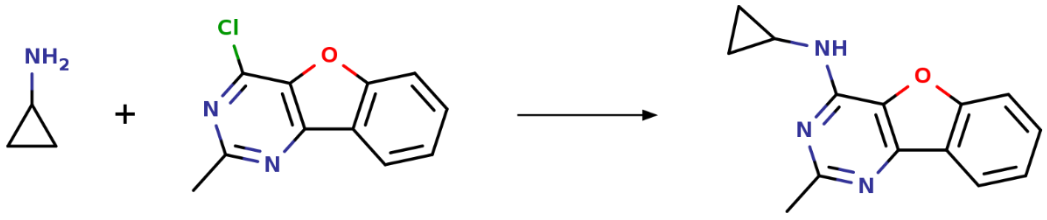 |
| CAS | 765-30-0;39786-40-8;N/A |
| Yield(%) | 100 |
| Similarity(%) | 36 |
| Conditions | DIPEA; 1,4-dioxane |
| Procedures | A solution of 4-chloro-2-methyl-benzofuro[3,2-djpyrimidine (70.0 mg, 0.32 mmol), DIEA (80.0 jiL, 0.467 mmol) and cyclopropylamine (70 jiL, 1.0 mol) in 1,4-dioxane (2.0 mL) was heated at 140 ° C ina microwave reactor for 4 h to give a pale yellow suspension. The solvent was evaporated in vacuo to afford an orange glassy residue. The crude residue was purified by reverse-phase preparative HPLC on a Biotage KP-C18-HS (120 g) column and a gradient 10-50% acetonitrile in water containing 0.05% TFA. The pure fractions were combined and evaporated in vacuo to afford a colorless to pale yellow viscous residue. The purified residue was dissolved in acetonitrile containing a trace of methanol andthe solution was passed through a SiliaPrep Carbonate (Si-CO3) 6 mL-1 g cartridge to |

| | |
|---|---|
| | neutralize TFA. The filtrate was evaporated in vacuo to afford a cream gummy solid (98.0 mg). The residue was dissolved in water containing a trace of acetonitrile and the solution was lyophilized to afford a cream lyophilized powder (85.7 mg, yield 100%). LC-MS analysis of the solid showed the desired product with a purity >98% and the desired product?s mass: m/z 240 (M+H); Calcd for C14H13 N3O: 239.28. 1HNMR (400 MHz, DMSO-d6): oe 0.64-0.71 (m, 2H, -CH2-), 0.75-0.85 (m, 2H, -CH2-), 2.57 (s, 3H, 2- CH3-), 3.11 (td, J = 7.23 and 3.82 Hz, 1H, -CH- (cypyl)), 7.45-7.5 1 (m, 1H), 7.64-7.71 (m, 1H), 7.71- 7.78 (m, 1H), 8.03-8.12 (m/d, 1H), 8.35 (brs,1H, -NH-). |
| **References** | WO2019/18359; A1; (2019) [13] |
| **DOI/Link** | N/A |
| **ID** | 11b (condition 3) |
| **ReferenceRXN** | 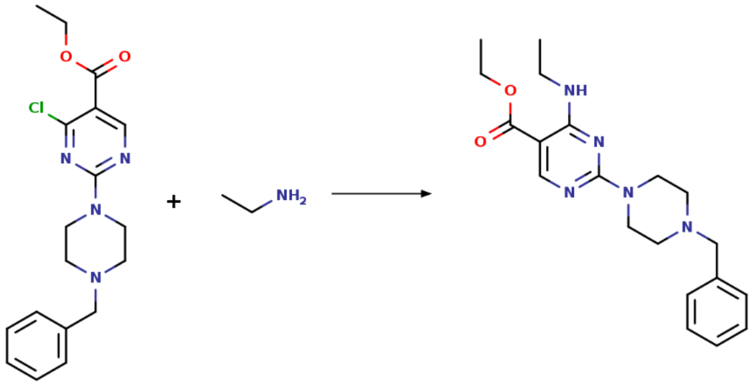 |

| | |
|---|---|
| **CAS** | 104966-24-7;75-04-7;N/A |
| **Yield(%)** | 100 |
| **Similarity(%)** | 38 |
| **Conditions** | EtNH2; CHCl3; H2O |
| **Procedures** | REFERENTIAL EXAMPLE 64 Ethyl 2-(4-benzylpiperazino)-4-ethylaminopyrimidine-5-carboxylate STR97 To a mixture of 101.3 g (0.28 mol) of ethyl 2-(4-benzylpiperazino)-4-chloropyrimidine-5-carboxylate (the compound synthesised in Referential Example 63) and 590 ml of chloroform, 72.3 g (1.12 mol) of a 70% aqueous solution of ethylamine was added dropwise over 15 minutes. The resultant mixture was stirred at room temperature for 2 hours. The reaction mixture was added with water, followed by extraction with chloroform. After drying the chloroform layer with MgSO4, chloroform was distilled off to obtain 103.5 g of the above-identified compound as an oily product (yield: about 100%). 1 H-NMR spectrum (CDCl3 solution, delta ppm): 1.22 (3H, t, J=7 Hz), 1.32 (3H, t, J=7 Hz), 2.47 (4H, m), 3.46 (2H, d, q, J=5, 7 Hz), 3.54 (2H, s), 3.88 (4H, m), 4.25 (2H, q, J=7 Hz), 7.31 (5H, s), 8.00 (1H, br. t, J=5 Hz), 8.58 (1H, s). |
| **References** | US4734418; A; (1988)[14] |
| **DOI/Link** | N/A |
| **ID** | 10a |

| | |
|---|---|
| ReferenceRXN | 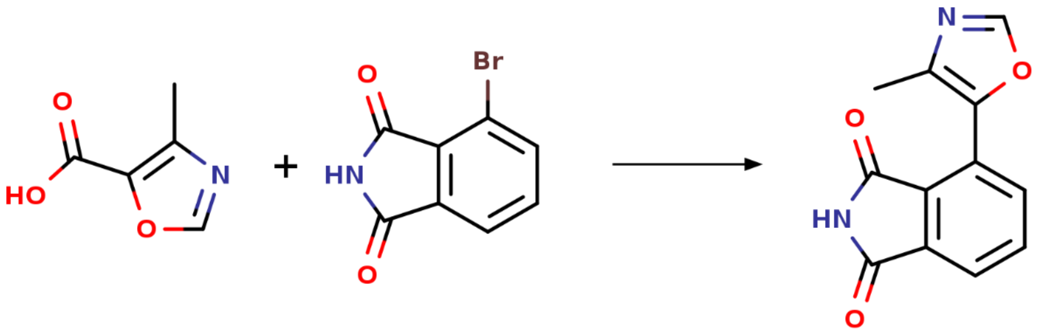 |
| CAS | 2510-32-9;70478-63-6;N/A |
| Yield(%) | 52 |
| Similarity(%) | 57 |
| Conditions | DMF; Cs2CO3; TBACl; Pd(t-Bu3P)2; Inert atmosphere; Microwave |
| Procedures | To a stirred mixture of 4-bromo-2H-isoindole-1,3-dione (50.00 mg, 0.221 mmol, 1.00 equiv), Cs2CO3 (108.11 mg, 0.332 mmol, 1.5 equiv) and 4-methyl-1,3-oxazole-5-carboxylic acid (56.23 mg, 0.442 mmol, 2 equiv) in DMF (4.00 mL) were added Pd(P(t-Bu)3)2 (5.65 mg, 0.011 mmol, 0.05 equiv) and tetrabutyl ammonium chloride (61.48 mg, 0.221 mmol, 1 equiv) in one portions at room temperature under nitrogen atmosphere. The final reaction mixture was irradiated with microwave radiation for 15 min at 170 oC. The reaction was monitored by LCMS. The mixture was allowed to cool down to room temperature. The residue was purified by reverse phase Flash chromatography with the following conditions: Column: WelFlash TM C18-I, 20-40 mm, 120 g; Eluent A: water (plus 10 mmol/L TFA); |

| | |
|---|---|
| | Eluent B: ACN; Gradient: 25% - 45% B in 25 min; Flow rate: 60 mL/min; Detector: 220/254 nm; desired fractions were collected at 38% B and concentrated under reduced pressure to afford 4-(4-methyl-1,3-oxazol-5-yl)-2H-isoindole-1,3- dione (26 mg, 52%) as an off-white solid. 1H NMR (400 MHz, DMSO-d6) d 11.43 (s, 1H), 8.46 (s, 1H), 7.95- 7.84 (m, 3H), 2.13 (s, 3H). LC/MS (ESI, m/z): [(M + 18)]+ = 229.05. |
| **References** | WO2021/011631; A1; (2021) [15] |
| **DOI/Link** | N/A |
| **ID** | 10b (condition 1) |
| **ReferenceRXN** | 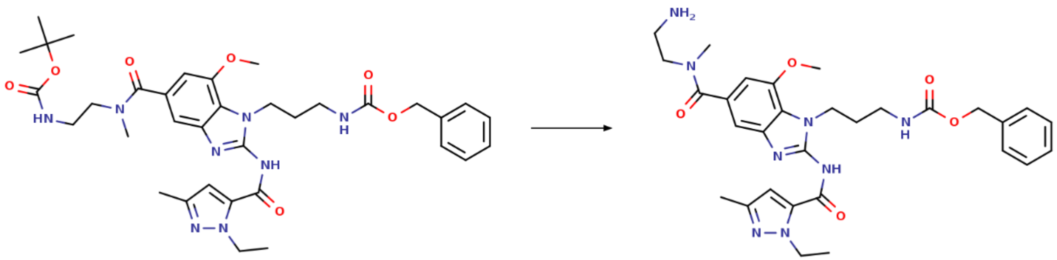 |
| **CAS** | N/A;N/A |
| **Yield(%)** | 21 |

| | |
|---|---|
| **Similarity(%)** | 74 |
| **Conditions** | DCM; TFA |
| **Procedures** | To a solution of benzyl N-(3-[5-[(2-[[(tert- butoxy)carbonyl]amino]ethyl)(methyl)carbamoyl]-2-(l-ethyl-3-methyl-lH-pyrazole-5-amido)-7- methoxy-lH-l,3-benzodiazol-l-yl]propyl)carbamate (120 mg, 0.174mmol, 1 equiv.) in dichloromethane (10 mL) was added trifluoroacetic acid (2 mL). The resulting mixture was stirred at RT overnight. The resulting mixture was concentrated under reduced pressure and basified with Sat. NaHCCb (aq.). The crude product was purified by Prep-HPLC with the following conditions: Column, Sunfire Prep C18 OBD Column 19* 150mm, 5um; mobile phase, water (0.1 percent FA) and ACN (15 percent ACN up to 34 percent in 8 min); UV detection at 254/220 nm. The product-containing fractions were collected and evaporated partially and lyophilized overnight to afford 22.2 mg (20.9 percent) of 1-64 as a white solid. (ES, m/z): 591 (M+H+); 1HNMR (400 MHz, DMSO-d6) d 8.35 (s, 1H), 7.40-7.21 (m, 6H), 7.10-6.90 (m, 1H), 6.67 (s, 1H), 5.01 (s, 2H), 4.60 (q, 2H), 4.36 (t, 2H), 3.93 (s, 3H), 3.70-3.20 (m, 3H), 3.14-3.02 (m, 2H), 3.01-2.79 (m, 4H), 2.90-2.78 (m, 1H), 2.14 (s, 3H), 1.99-1.90 (m, 2H), 1.35 (t, 3H). |
| **References** | WO2020/132566; A1; (2020) [2] |
| **DOI/Link** | N/A |
| **ID** | 10b (condition 2) |

| ReferenceRXN | 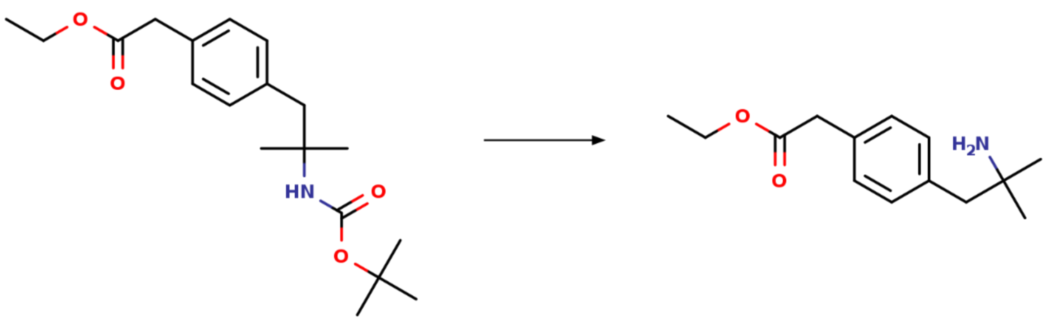 |
| --- | --- |
| CAS | 1251957-73-9;808768-89-0 |
| Yield(%) | 100 |
| Similarity(%) | N/A |
| Conditions | H2O; HCl; K2CO3; 1,4-dioxane; EtOH |
| Procedures | # Synthesis of the Ethyl [4-(2-Amino-2-methylpropyl)phenyl]acetate□□HCl (4 M) in dioxane (30 mL) was added to a stirred solution of the ester 5 (3.75 g, 11.18 mmol) in ethanol (30 mL) and the mixture stirred at room temperature overnight. The reaction mixture was evaporated in Vacuo and 10% aqueous potassium carbonate added until the pH of the mixture reached 8. The mixture was extracted with ethyl acetate (3 150 mL), and the combined extracts were washed with brine, dried over MgSO4, and evaporated in Vacuo to give the title compound 6 as a yellow oil (2.72 g, 100%). 1H NMR (400 MHz, CDCl3) δ: 1.10 (s, 6H), 1.24 (t, J ) 7.2 Hz, 3H) 2.64 (s, 2H), 3.58 (s, 2H), 4.16 (q, J ) 7.2 Hz, 2H), 7.15 7 (br d, J ) 8.2 Hz, 2H), 7.22 (br d, J ) 8.2 Hz, 2H); LC/MS Rt ) 0.73 min, m/z |

| | |
|---|---|
| | (ES+) 236 [MH+]. |
| **References** | Organic Process Research and Development; vol. 14; 6; (2010); p. 1326 - 1336 [3] |
| **DOI/Link** | [10.1021/op1001462](https://doi.org/10.1021/op1001462) |
| **ID** | 10b (condition 3) |
| **ReferenceRXN** | 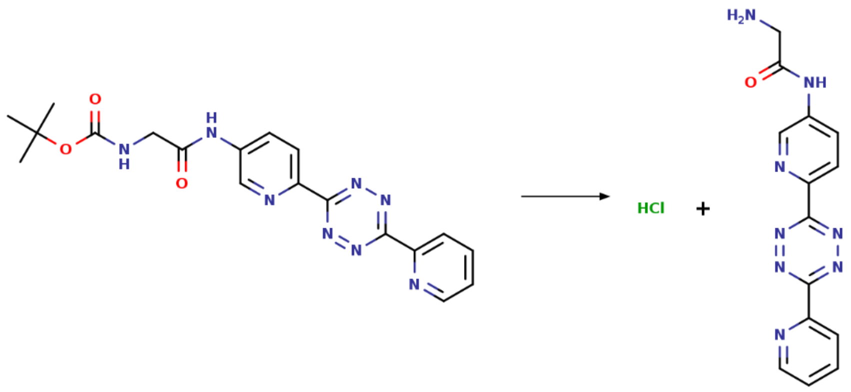 |
| **CAS** | 1360467-31-7;7647-01-0;1360535-79-0 |
| **Yield(%)** | 100 |
| **Similarity(%)** | 27 |

| | |
|---|---|
| **Conditions** | HCl; 1,4-dioxane; DCM |
| **Procedures** | Boc-protected Tetrazine S6 was synthesized using the procedure reported earlier6. 4M HCl in dioxane (500 mu,, 2,0 mmol) was added to a stirring solution of Tetrazine S5 (8 mg, 0.02 mmol) in DCM (500 muL). The reaction was carried out for 2 h at room temperature and subsequently the solvent was removed under reduced pressure to yield primary amine hydrochloride S6 as a pink solid (6mg, 0.02 mmol, 100 percent). The compound was directly used in the next step without any further purification. |
| **References** | WO2015/136265; A1; (2015)[4] |
| **DOI/Link** | N/A |
| **ID** | 8a (condition 1) |
| **ReferenceRXN** | 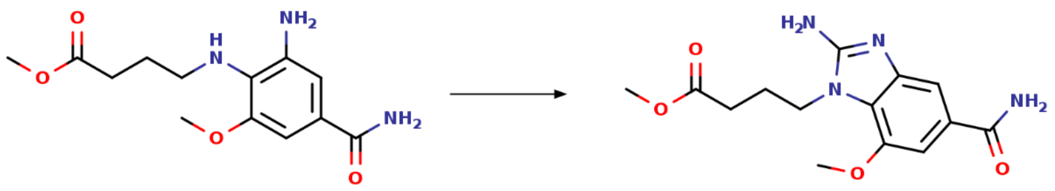 |
| **CAS** | N/A;N/A |

| | |
|---|---|
| **Yield(%)** | 53 |
| **Similarity(%)** | 68 |
| **Conditions** | MeOH; Inert atmosphere |
| **Procedures** | General procedure: After compound 1-4a (4 g, 11.33 mmol) was dissolved in methanol, cyanogen bromide (2.26 g, 21.52 mmol) was added, and under the protection of nitrogen, the temperature was raised to 60 C. to react overnight. After the reaction was complete, cool to room temperature, concentrate to remove volatile solvent, add saturated K2CO3solution and stir, a solid precipitated, filtered, the obtained white solid was washed with ether, and dried in vacuo to obtain compound 1-5a with a yield of 70 %. |
| **References** | CN115141182; A; (2022) [16] |
| **DOI/Link** | N/A |
| **ID** | 8a (condition 2) |

| | |
|---|---|
| **ReferenceRXN** | 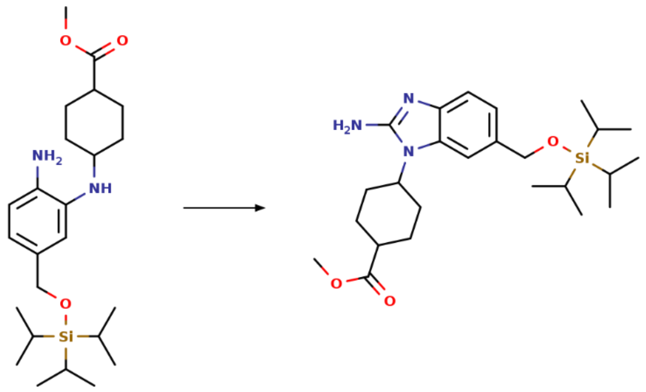 |
| **CAS** | N/A;N/A |
| **Yield(%)** | 100 |
| **Similarity(%)** | 54 |
| **Conditions** | NaOH; DCM; EtOH; H2O |
| **Procedures** | cis-Methyl 4-(2-amino-6-((triisopropylsilyloxy)methyl)-lH- benzo[d]imidazol-l-yl)cyclohexanecarboxylate. A round bottom flask under nitrogen atmosphere was charged with cis-methyl 4-(2-amino-5-((triisopropylsilyloxy)methyl)phenylamino)cyclohexanecarboxylate (2.5 g, 5.75 mmol) and EtOH (23.96 mL) To this was added cyanogen bromide (0.914 g, 8.63 mmol), and the reaction mixture was stirred overnight at RT. The reaction mixture was diluted with DCM (80 mL) and washed with lN aqueous NaOH (50mL). The organic layer was collected, dried over sodium sulfate, and concentrated to dryness to afford cis-methyl 4- (2-amino-6-((triisopropylsilyloxy)methyl)-lH-benzo[d]imidazol-l- yl)cyclohexanecarboxylate as a brown solid (2.64 g, 100 percent yield). MS |

| | |
|---|---|
| | m/z = 460.2 [M+H]. Calc'd for C25 |
| **References** | WO2012/18668; A1; (2012) [17] |
| **DOI/Link** | N/A |
| **ID** | 7a (condition 1) |
| **ReferenceRXN** | 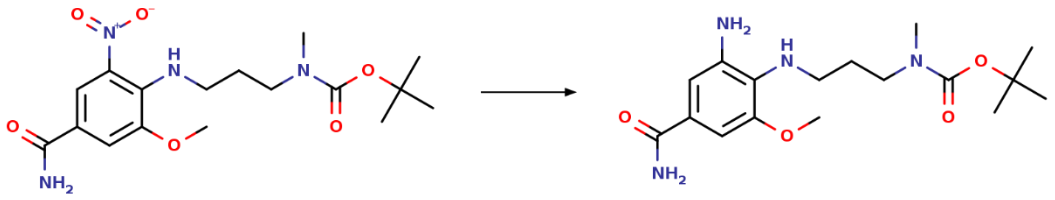 |
| **CAS** | N/A;N/A |
| **Yield(%)** | N/A |
| **Similarity(%)** | 99 |

| | |
|---|---|
| **Conditions** | MeOH; THF; H2; Pd/C |
| **Procedures** | N/A |
| **References** | Journal of Medicinal Chemistry; vol. 66; 8; (2023); p. 5584 - 5610 [9] |
| **DOI/Link** | 10.1021/acs.jmedchem.2c02046 |
| **ID** | 7a (condition 2) |
| **ReferenceRXN** | 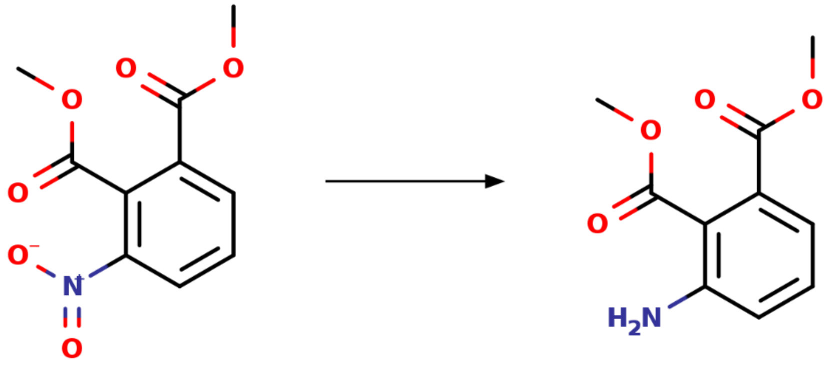 |
| **CAS** | 13365-26-9;34529-06-1 |
| **Yield(%)** | 100 |

| | |
|---|---|
| **Similarity(%)** | 29 |
| **Conditions** | Pd/C; H2; MeOH |
| **Procedures** | [0030] The synthesis of mIBX is readily accomplished from commercially available 3-nitrophtalic acid as follows: esterification of 3-nitrophtalic acid via the corresponding acid chloride to give nitrodiester (100 percent), which upon catalytic hydrogenation provides the aminodiester (100 percent). Diazotization is then performed, followed by iodination of the aminodiester to provide dimethyl 3-iodophthalate in about 91 percent yield. This is followed by saponification, then acidification of dimethyl 3-iodophthalate to give 3-iodophthalic acid in about 93 percent yield. 3-iodophthalic acid is then oxidized to form the water-soluble MIBX. This process is carried out using KBrO3 in 0.73H2SO4 at 55-60° C. as follows: KBrO3 (5 g, 30 mmol) is added in portions to a suspension of 3-iodophthalic acid (5 g, 17.1 mmol) in 70 ML of 0.73 M H2SO4 over a period of 20 minutes; The mixture is then maintained at 55-60° C. for 12 hours and the resulting clear orange solution is evaporated to yield an off-white solid, which is triturated with 30 ML of water at 0° C. for 2 hours and filtered to obtain a white solid. This is further triturated with hexane (100 ML) for 6 hours and filtered to give MIBX (3.9 g, 71 percent) as a white solid with a melting point of 258-260° C. The approximately 70 percent yield for the conversion of 3-iodophthalic acid to MIBX is the isolated yield of MIBX, with the actual conversion near quantitative as evident from monitoring the oxidation of 3-iodophthalic acid to MIBX by 1H NMR spectroscopy. Water-soluble MIBX is isolated as an analytically pure white solid. The synthesis of MIBX from 3-nitrophthalic acid is illustrated in . The physical properties of MIBX are as follows: mp 258-260° C.; IR (KBr), 3503 3469, 3050, 1708, 1631, 1588, 1369, 730, 700 cm-1; 1H NMR (D2O), 300 MHz): delta 8.35 (dd, J=7.9, 1.0 Hz, 1H), 8.09 (t, J=7.9 Hz, 1H), 7.94 (dd, J=7.9, 1.0 Hz, 1H); 13C NMR (D2O, 75 MHz): delta 125.5, 127.5, 132.5, 134.7, 137.0, 147.1 (ring carbons), 168.9, 172.9 (carbonyl carbons). |
| **References** | US2004/30187; A1; (2004) [18] |

| | |
|---|---|
| DOI/Link | N/A |
| ID | 7a (condition 3) |
| ReferenceRXN | 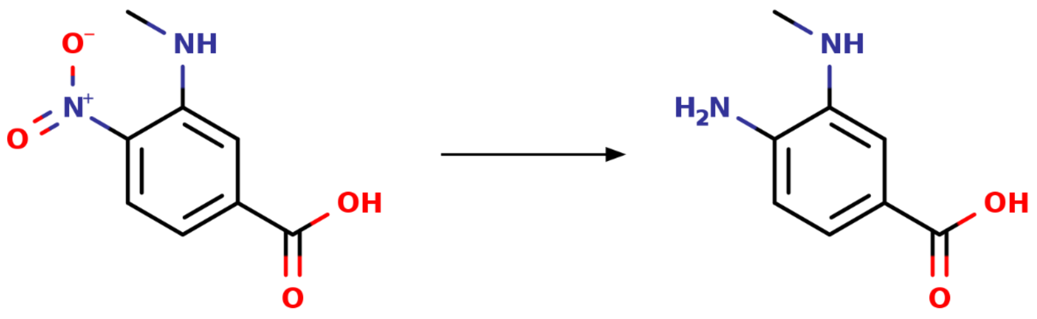 |
| CAS | 214778-10-6;66630-74-8 |
| Yield(%) | 100 |
| Similarity(%) | 32 |
| Conditions | Raney Ni; H2 |
| Procedures | N/A |

| ID | 6a (condition 1) |
| --- | --- |
| ReferenceRXN | 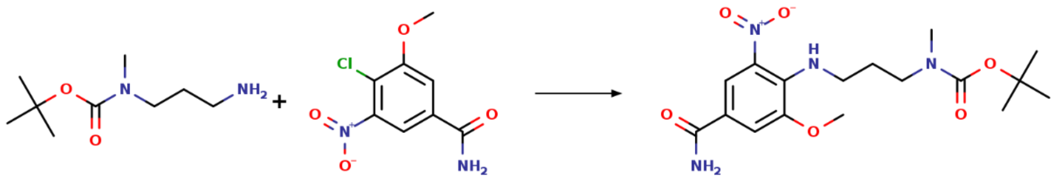 |
| CAS | 150349-36-3;2137975-62-1;N/A |
| Yield(%) | 57 |
| Similarity(%) | 74 |
| Conditions | DMF; K2CO3; Inert atmosphere |
| Procedures | Into a 100-mL round-bottom flask purged and maintained with an inert atmosphere of nitrogen, was placed 1.1 (3.00 g, 13.010 mmol, 1.00 eq), tert-butyl N-(3-aminopropyl)-N-methylcarbamate (2.94 g, 0.016 mmol, 1.20 eq) and K2CO3 (5.39 g, 0.039 mmol, 3.00 eq) in DMF (30.00 mL). The solution was stirred for 12 h at 70Â° C in an oil bath. The reaction was then quenched by the addition of 30 mL of water. The resulting solution was extracted with 3x30 mL of ethyl acetate. The resulting mixture was washed with 1 x30 ml of |

| | |
|---|---|
| | brine. The solid was dried in an oven under reduced pressure. The solids were filtered out. The resulting mixture was concentrated. The residue was applied onto a silica gel column with ethyl acetate/petroleum ether (1 : 1). This resulted in 2.8 g (56.28 percent) of 48.1 as a yellow solid. (ES, m/z): 383 (M+H)+. |
| **References** | WO2020/132566; A1; (2020) [2] |
| **DOI/Link** | N/A |
| **ID** | 6a (condition 2) |
| **ReferenceRXN** | 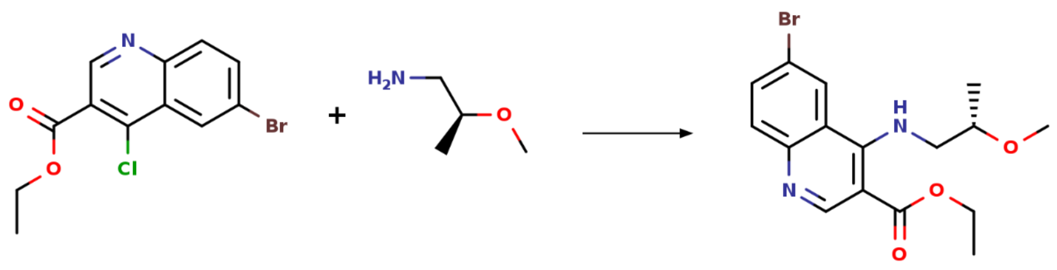 |
| **CAS** | 206257-39-8;907943-71-9;1386874-27-6 |
| **Yield(%)** | 100 |

| | |
|---|---|
| **Similarity(%)** | 36 |
| **Conditions** | DIPEA; EtOH |
| **Procedures** | Suspend ethyl-6-bromo-4-chloro-quinoline-3-carboxylate (389 g, 1.24 mol) and (2S)-2-methoxypropan-1-amine, hydrochloride (171 g, 1.36 mol, 1.1 eq.) in ethanol (5.84 L). Add diisopropylethylamine (474 mL) and heat the mixture at 50° C. overnight. After 16 hours, cool the reaction to room temperature and concentrate in vacuo. Add methyl tert-butyl ether (2 L) to the residue and stir for 20 min. Filter the precipitate and wash it with methyl tert-butyl ether (2*250 mL). Concentrate the filtrate in vacuo to afford the titled compound in almost quantitative yield. The compound will be used in the next step without further purification. MS (ESI) m/z (M+H)+367.0, 369.0 |
| **References** | US2012/184577; A1; (2012) [19] |
| **DOI/Link** | N/A |
| **ID** | 6a (condition 3) |

| | |
|---|---|
| **ReferenceRXN** | 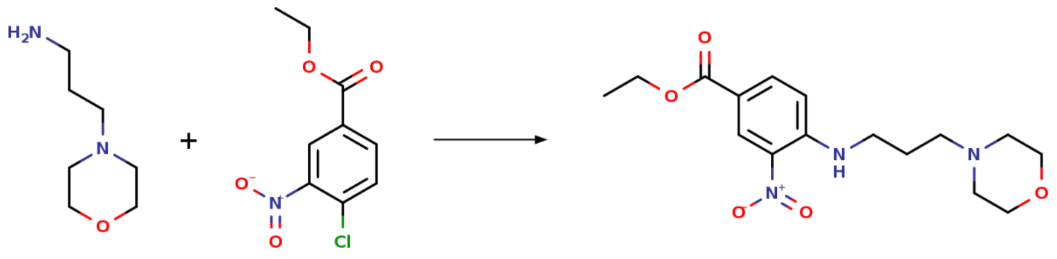 |
| **CAS** | 123-00-2;16588-16-2;310451-70-8 |
| **Yield(%)** | 100 |
| **Similarity(%)** | 51 |
| **Conditions** | TEA; EtOH |
| **Procedures** | A mixture of f-1 (0.022 mol), f-2 (0.066 mol) and NEt3 (0.066 mol) in C2H5OH (50 ml) was stirred at 60C for 12 hours, then evaporated to dryness and taken up in CH2Cl2. EPO <DP n="32"/>The organic layer was washed with H2O, dried (over MgSO4), filtered and the solvent was evaporated to dryness. Yield: 7.4 g of f-3 (100%). |
| **References** | W02006/97534; A1; (2006) [20] |

| DOI/Link | N/A |
|---|---|
| ID | 5b (condition 1) |
| ReferenceRXN | 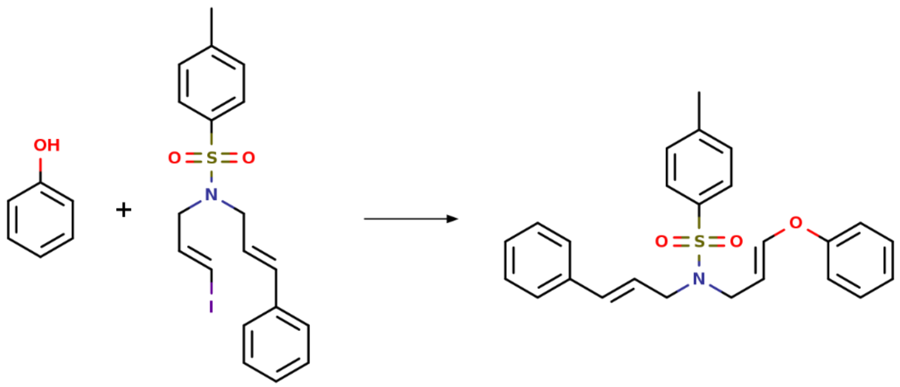 |
| CAS | 108-95-2;N/A;N/A |
| Yield(%) | 45 |
| Similarity(%) | 95 |
| Conditions | PhMe; Cs2CO3; NMM; CuCl; Reflux |
| Procedures | N/A |

| | |
|---|---|
| **References** | Angewandte Chemie, International Edition; vol. 51; 41; (2012); p. 10329 - 10332,4; Angewandte Chemie; vol. 124; 41; (2012); p. 10475 - 10478,4 [21] |
| **DOI/Link** | [10.1002/anie.201204835](https://doi.org/10.1002/anie.201204835) |
| **ID** | 5b (condition 2) |
| **ReferenceRXN** | 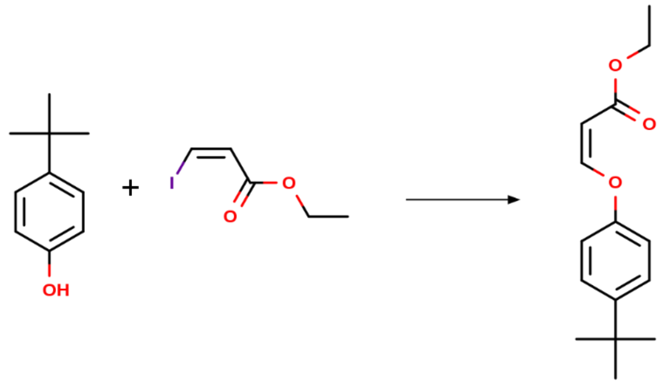 |
| **CAS** | 98-54-4;31930-36-6;N/A |
| **Yield(%)** | N/A |
| **Similarity(%)** | 27 |
| **Conditions** | nickel(II) bromide dimethoxyethane; 4CzIPN; MeO-bpy; TBACl; TMG; 1,4-dioxane |

| | |
|---|---|
| **Procedures** | 1. Under nitrogen atmosphere in a glovebox, add NiBr2・glyme (0.010 mmol, 3.09 mg, 10 mol%), d(OMe) (4,4'-dimethoxy-2,2'-bipyridine, 0.015 mmol, 3.24 mg, 15 mol%), TBAC (0.020 mmol, 5.56 mg, 20 mol%) and TMG (0.15 mmol, 18.78 μL, 1.5 equiv.) to a 12 mL screw vial, and dissolve in 3 mL of 1,4-dioxane. 2. Add a phenol derivative (0.15 mmol, 1.5 equiv.), an alkenyl halide (0.10 mmol, 1.0 equiv.) and 4CzIPN (0.003 mmol, 2.37 mg, 3.0 mol%) to the mixture. 3. Cap the vial using a screw cap and seal with plastic tape. 4. Remove the vial containing the reaction mixture from the glovebox and place 10 cm in front of a 427 nm Kessil LED lamp (A160WE Tuna Blue); maintain the temperature of the reaction vials at ca. 25° C. 5. Irradiate with LED for 20 h. 6. Wash the reaction mixture with saturated NaHCO3 (aq), brine, and extract with AcOEt. 7. Concentrate in vacuo. 8. Purify the residue by flash chromatography on silica gel with the solvent combinations listed under TLC information in the "Characterization Data" below to give the product. |
| **ID** | 4b (condition 1) |
| **ReferenceRXN** | 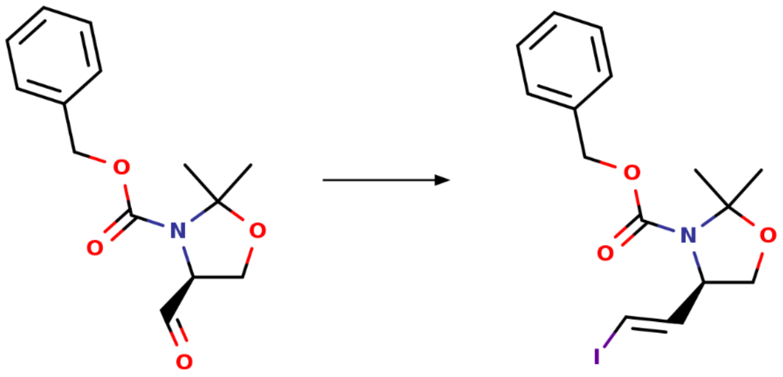 |
| **CAS** | 117833-92-8;N/A |

| Yield(%) | 56 |
| --- | --- |
| Similarity(%) | 60 |
| Conditions | THF; CrCl2 |
| Procedures | N/A |
| References | Journal of Organic Chemistry; vol. 67; 6; (2002); p. 1802 - 1815 [22] |
| DOI/Link | 10.1021/jo010865a |
| ID | 4b (condition 2) |
| ReferenceRXN | 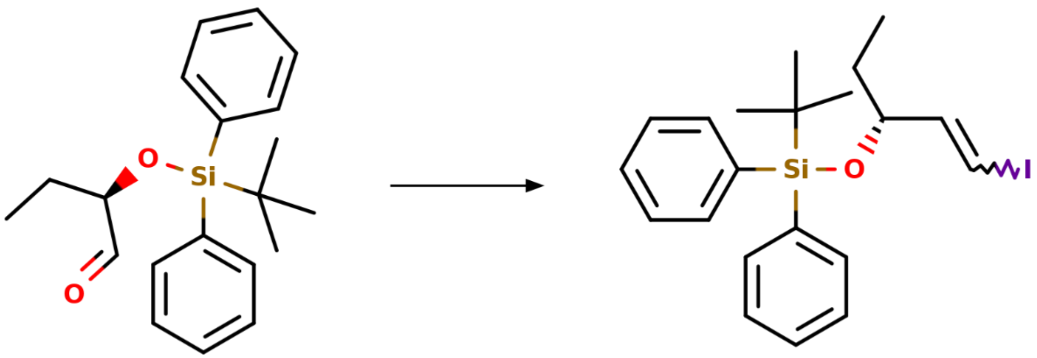 |

| | |
|---|---|
| **CAS** | 1309610-38-5;1309610-39-6 |
| **Yield(%)** | 70 |
| **Similarity(%)** | 29 |
| **Conditions** | CrCl2; THF |
| **Procedures** | N/A |
| **References** | Organic Process Research and Development; vol. 17; 6; (2013); p. 915 - 920 [23] |
| **DOI/Link** | 10.1021/op4000384 |
| **ID** | 4b (condition 3) |

| | |
|---|---|
| ReferenceRXN | 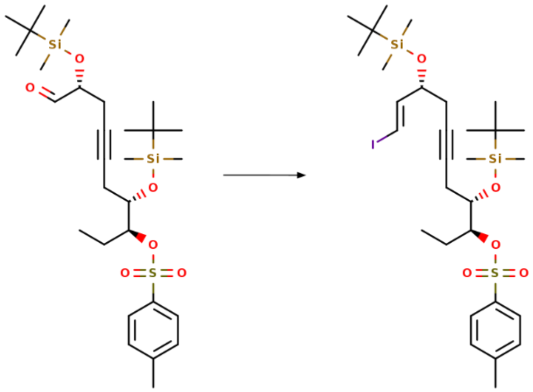 |
| CAS | N/A;N/A |
| Yield(%) | 88 |
| Similarity(%) | 30 |
| Conditions | CrCl2; CHI3; 1,4-dioxane; THF |
| Procedures | General procedure: According to the synthetic procedure of C10-20 fragment 3aa, 3bb (613 mg, 0.868 mmol) was synthesized from aldehyde 20bb (577 mg, 0.990 mmol) in 88 percent yield by using CrCl2 (836mg, 6.80 mmol) and iodoform (903 mg, 2.29 mmol) in a mixture of THF (4.0 mL) and 1,4-dioxane (12.4 mL). Purification was performed by flash column chromatography on silica gel (30 g, hexane to hexane/CH2Cl2 1/1): colorless oil; [alpha]D24 -41 (c 1.0, CHCl3); IR (neat) nu 2953, 2929, 2856, 1917, 1600,1463, 1363, 1255, 1188, 1177, 1098, 931 cm-1; 1H NMR (400 MHz, CDCl3) delta 0.05 (3H, s, CH3 of TBS), 0.06 (3H, s, CH3 of TBS), 0.07 (3H, s, CH3 of TBS), 0.08 (3H, s, CH3 of TBS), 0.76 (3H, t, J=7.4 Hz, H20), 0.87 (9H, s, t-Bu of TBS), 0.89 (9H, s, t-Bu of TBS), 1.51 (1H, |

| | |
|---|---|
| | m, H19a), 1.75 (1H, m, H19b), 2.16-2.40 (4H, m, H13 and H16), 2.45 (3H, s, CH3 of Ts), 3.91 (1H, dt, J=8.7, 4.5 Hz, H17), 4.19 (1H, m, H12), 4.35 (1H, dt, J=8.7, 4.1 Hz, H18), 6.31 (1H, dd, J=14.6, 1.4 Hz, H10), 6.66 (1H, dd, J=14.6, 5.5 Hz, H11), 7.34 (2H, d, J=8.7 Hz, aromatic), 7.80 (2H, d, J=8.7 Hz, aromatic); 13C NMR (100 MHz, CDCl3) delta -4.94, -4.88, -4.81, -4.6, 10.2, 17.9, 18.2, 21.3, 21.6, 22.2, 25.68 (*3), 25.73 (*3), 28.2, 71.4, 73.9, 76.6, 77.6, 79.5, 85.5, 127.8 (*2), 129.7 (*2), 134.1, 144.6, 147.5; HRMS (ESI) calcd for C30H51IO5SSi2Na 729.1933 [M+Na]+, found 729.1923. A solution of iodoform (676 mg, 1.72 mmol) and aldehyde 20aa (431 mg, 0.739 mmol) in 1,4-dioxane (4.4 mL) was added to a suspension of CrCl2 (629 mg, 5.11 mmol) in a mixture of THF (2.9 mL) and 1,4-dioxane (4.4 mL) at room temperature. The reaction mixture was stirred at room temperature for 17 h, and then H2O (5 mL) was added. The resultant mixture was extracted with Et2O (15 mL * 3), and the combined organic layers were washed with H2O (10 mL) and brine (10 mL), dried over Na2SO4, filtered, and concentrated. The residue was purified flash column chromatography on silica gel (25 g, hexane to hexane/CH2Cl2 1/1) to afford C10-20 fragment 3aa (389 mg, 0.551 mmol) in 75 percent yield. |
| **References** | Tetrahedron; vol. 71; 43; (2015); p. 8320 - 8332 [24] |
| **DOI/Link** | 10.1016/j.tet.2015.08.047 |
| **ID** | 3a (condition 1) |

| | |
|---|---|
| **ReferenceRXN** | 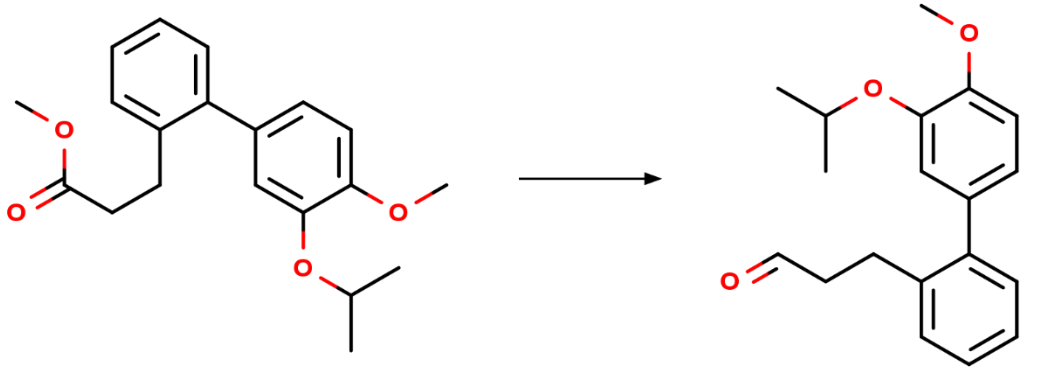 |
| **CAS** | 1624332-60-0;1624332-77-9 |
| **Yield(%)** | 100 |
| **Similarity(%)** | 23 |
| **Conditions** | PhMe; n-hexane; DIBAL-H |
| **Procedures** | N/A |
| **References** | Chemistry - A European Journal; vol. 20; 22; (2014); p. 6752 - 6755 [25] |
| **DOI/Link** | [10.1002/chem.201402015](10.1002/chem.201402015) |

| ID | 3a (condition 2) |
|---|---|
| ReferenceRXN | 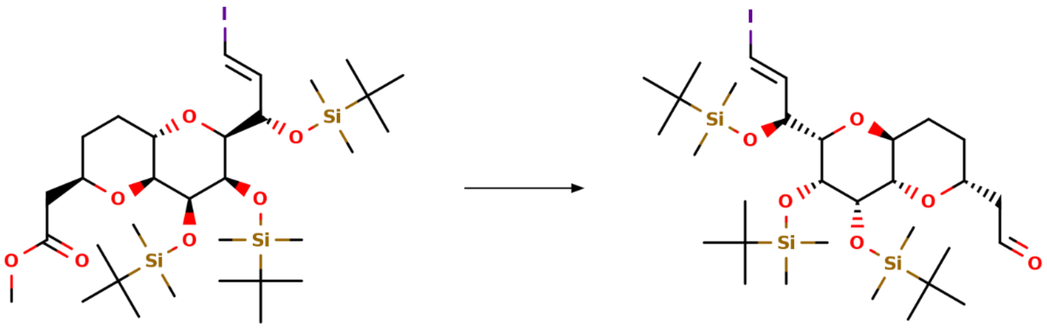 |
| CAS | N/A;N/A |
| Yield(%) | 100 |
| Similarity(%) | 27 |
| Conditions | 2,6-Di-tert-butyl-4-methylphenol; DIBAL-H; PhMe |
| Procedures | N/A |
| References | Synlett; vol. 24; 3; (2013); p. 333 - 337 [26] |

| | |
|---|---|
| DOI/Link | 10.1055/s-0032-1318026 |
| ID | 3a (condition 3) |
| ReferenceRXN | 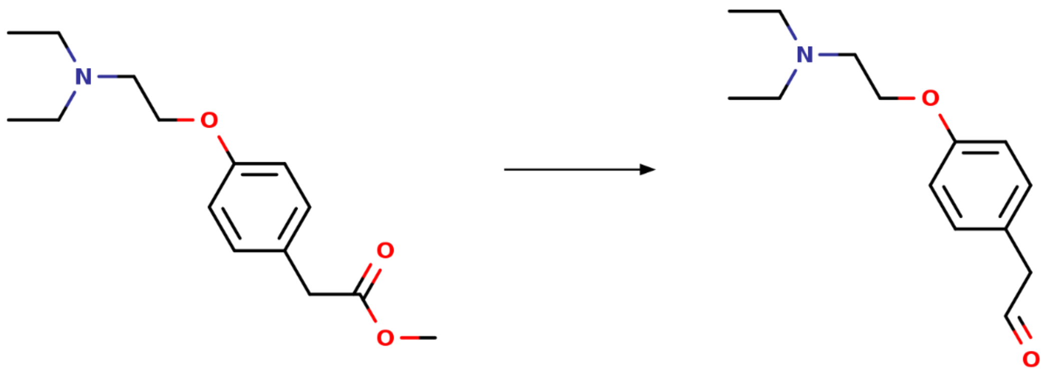 |
| CAS | 845889-08-9;845889-09-0 |
| Yield(%) | 100 |
| Similarity(%) | 29 |
| Conditions | DIBAL-H; PhMe |
| Procedures | {4-[2-(Diethylamino)ethoxy]phenyl} acetaldehyde To a stirred solution of methyl {4- [2- (diethylamino) ethoxy] phenyl} acetate (5.3 g, 20 mmol) in anhydrous toluene (100 mL) cooled to -78 ° C under N2 was added diisobutylaluminum hydride (DIBAL, 1. 0M in |

| | |
|---|---|
| | toluene, 100 mL, 100 mmol) over a period of 10-15 minutes. The mixture was stirred at-78 ° C for an additional 2h. The reaction was quenched by the slow addition of MeOH, followed by the introduction of 10 percent Rochelle's Salt. The suspension was diluted with EtOAc and stirred at room temperature for LH. The EtOAc layer was set aside and the aqueous layer was extracted with EtOAc (2x). The combined organic layers were combined and dried over NA2SO4 and filtered. The solution was concentrated under vacuum to yield 4.7 g (100 percent) of the title aldehyde as a yellow viscous semisolid, which was used in the next step without purification. |
| **References** | W02005/16909; A1; (2005) [20] |
| **DOI/Link** | N/A |
| **ID** | 2a (condition 1) |
| **ReferenceRXN** | 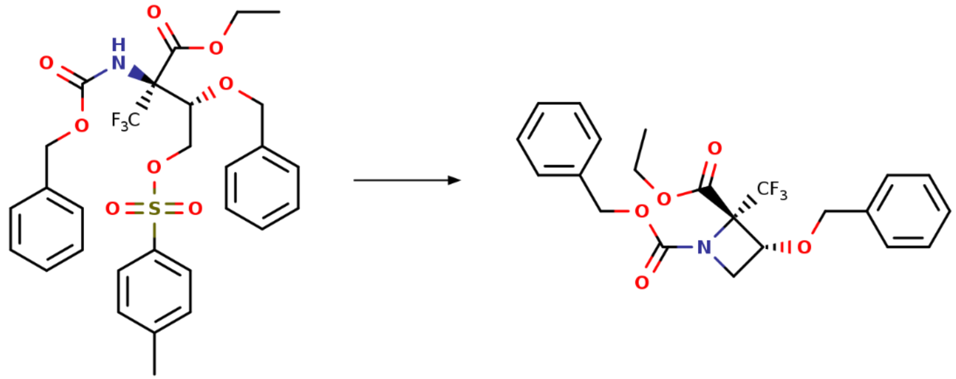 |
| **CAS** | N/A;N/A |

| | |
|---|---|
| **Yield(%)** | 85 |
| **Similarity(%)** | 57 |
| **Conditions** | THF; n-hexane; n-BuLi |
| **Procedures** | N/A |
| **References** | Journal of Organic Chemistry; vol. 64; 23; (1999); p. 8731 - 8735 [27] |
| **DOI/Link** | 10.1021/jo9909397 |
| **ID** | 2a (condition 2) |
| **ReferenceRXN** | 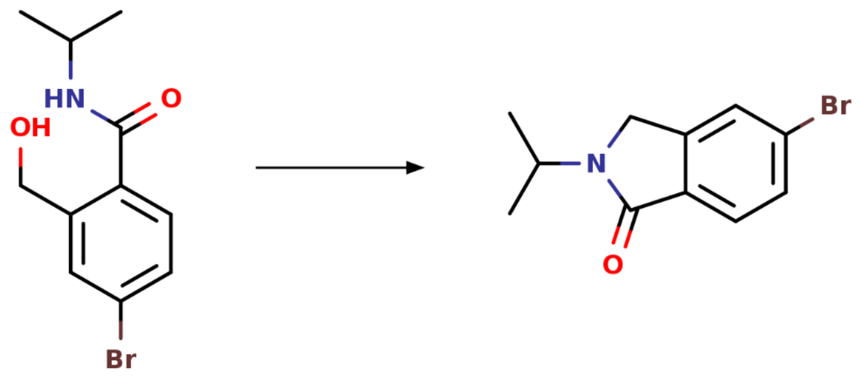 |

| | |
|---|---|
| **CAS** | 888030-75-9;864866-47-7 |
| **Yield(%)** | 64 |
| **Similarity(%)** | 24 |
| **Conditions** | DEPC; i-PrMgCl; DMEU; heptane; i-PrOH |
| **Procedures** | N/A |
| **References** | Organic Process Research and Development; vol. 13; 6; (2009); p. 1407 - 1412 [28] |
| **DOI/Link** | 10.1021/op900062p |
| **ID** | 2a (condition 3) |

| | |
|---|---|
| ReferenceRXN | 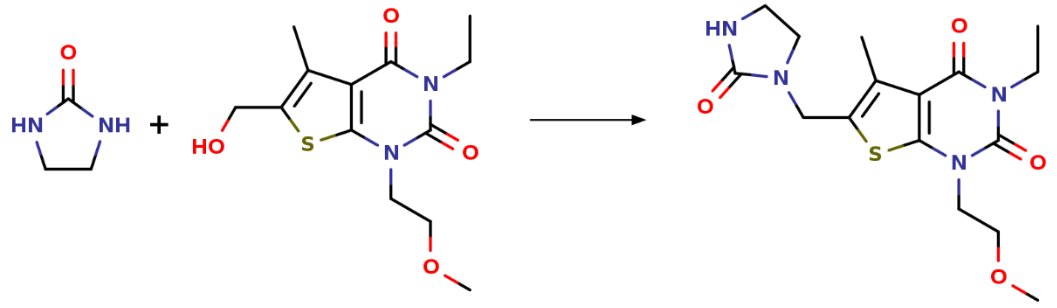 |
| CAS | 120-93-4;N/A;N/A |
| Yield(%) | 60 |
| Similarity(%) | 27 |
| Conditions | DIPEA; NaH; SOCl2; DCM; THF; mineral oil |
| Procedures | Method A: To a solution of 99 mg (1.15 mmol) of imidazolidin-2-one in 3 ml of THF were added 46 mg (1.15 mmol) of sodium hydride (60 percent suspension in mineral oil), and the mixture was heated to 60° C. for 2 h and subsequently cooled back down to RT ("Solution 1"). To a solution of 90 mg (0.287 mmol) of the compound from Ex. 143A in 2 ml of dichloromethane in another reaction vessel were added, at 0° C., 100 mul (0.573 mmol) of N, N-diisopropylethylamine and 22 mul (0.301 mmol) of thionyl chloride. After 20 min at 0° C., Solution 1 was added dropwise and then the cooling bath was removed. The reaction mixture was stirred at RT for about 18 h. Then all the volatile constituents were removed on a rotary evaporator. The remaining residue was separated into its |

| | |
|---|---|
| | components by means of preparative HPLC (Method 8). After concentration of the product fractions and drying under high vacuum, 63 mg (60 percent of theory) of the title compound were obtained. |
| **References** | US2018/65981; A1; (2018) [29] |
| **DOI/Link** | N/A |


[1] Kim H., Lee K., Kim C., Lim J., Kim W. Y. DFRscore: Deep Learning-Based Scoring of Synthetic Complexity with Drug-Focused Retrosynthetic Analysis for High-Throughput Virtual Screening. J Chem Inf Model. 2024;64(7):2432-2444.

[2] NIMBUS TITAN, INC. STING Pyrazole Agonists and Uses Thereof. WO 2020132566 A1, 2020, World Intellectual Property Organization.

[3] Bradley, P. A.; Carroll, R. J.; Lecouturier, Y. C.; Moore, R.; Noeureuil, P.; Patel, B.; Wheeler, S. The synthesis of two potent β-3 adrenergic receptor agonists. Org. Process Res. Dev. 2010, 14, 1326-1336.

[4] MEDICAL RESEARCH COUNCIL. Cyclopropene Amino Acids and Methods. WO 2015136265 A1, 2015, World Intellectual Property Organization.

[5] Parker, J. S.; McCormick, M.; Anderson, D. W.; Maltman, B. A.; Gingipalli, L.; Toader, D. The development and scale-up of an antibody drug conjugate tubulysin payload. Org. Process Res. Dev. 2017, 21, 1602-1609.

[6] Li, J.; Zhang, D. Y.; Pan, F.; Ma, R.; Zhu, W. J.; Lü, K. Z.; Chen, X.; Zhang, Y. A macrocyclic immunomodulator. CN 111471056 A, 2020, China National Intellectual Property Administration.

[7] Moseley, J. D.; Moss, W. O.; Welham, M. J. Investigation of an alternative route to ZD3638 and cost-benefit analysis comparison of raw materials with the previous route. Org. Process Res. Dev. 2001, 5, 491-497.

[8] Bonrath, W.; Bourgeois, F.; Medlock, J. A.; Sparr, C. Stereoselective Synthesis of Enantiomerically-Enriched Pantolactone. WO 2019228874 A1, 2019, World Intellectual Property Organization.

[9] Chen, N. N.; Zhang, H.; Zhu, Q. S.; Zeng, T.; Dai, W.; Zhou, Y. L.; Xin, G. F.; Wu, B. D.; Gong, S. J.; Jiang, Z. Y.; You, Q. D.; Xu, X. L. Development of Orally Bioavailable Amidobenzimidazole Analogues Targeting Stimulator of Interferon Gene (STING) Receptor. J. Med. Chem. 2023, 66, 5584-5610.

[10] Rowbottom, M. W.; Hutchinson, J. H. Lysyl Oxidase-Like 2 Inhibitors and Uses Thereof. WO 2017003862 A1, 2017, World Intellectual Property Organization.

[11] Kim, D. S.; Yoshizawa, K.; Mitasev, B.; Schnaderbeck, M.; Zhang, H.; Omori, M.; Kayano, A.; Nagai, M.; Wakasugi, K.; Watanabe, Y.; Benayoud, F. Synthesis of BACE1 Inhibitors E2609/E2071 via Oxime–Olefin Cycloaddition Following a Process Risk Mitigation Strategy. Org. Process Res. Dev. 2021, 26, 804-816.

[12] Yasuma, T.; Sasaki, S.; Sakai, N. Phenylpropanoic Acid Derivatives. EP 1698624 A1, 2006, European Patent Office.

[13] Meyers, M. J.; Singh, M.; Stallings, C. L.; Weiss, L. A.; Wildman, S.; Arnett, S. D. Thieno[2,3-d]pyrimidines and Benzofuro[3,2-d]pyrimidines as Antimicrobial Agents. WO 2019018359 A1, 2019, World Intellectual Property Organization.

[14] Yokoyama, K.; Kato, K.; Kitahara, T.; Ohno, H.; Nishina, T.; Kumakura, M.; Awaya, A.; Nakano, T.; Watanabe, K.; Saruta, S. Quinazoline compounds and antihypertensives. US 4734418 A, 1988, United States Patent and Trademark Office.

[15] Ji, N.; Sintchak, M. D.; Zhang, Y.; Zheng, X. Fused-Glutarimide CRBN Ligands and Uses Thereof. WO 2021011631 A1, 2021, World Intellectual Property Organization.

[16] You, Q. D.; Xu, X. L.; Chen, N. N.; Zhang, H.; Zhu, Q. S.; Dai, W.; Zhou, Y. L.; Jiang, Z. Y.; Guo, X. K.; Wang, L.; Jin, Y. H. A benzimidazole compound, a pharmaceutical composition comprising the benzimidazole compound, and the use of both. CN 115141182 A, 2022, China National Intellectual Property Administration.

[17] Bode, C. M.; Cheng, A. C.; Choquette, D.; Lewis, R. T.; Potashman, M. H.; Romero, K.; Stellwagen, J. C.; Whittington, D. A. Benzimidazole and Azabenzimidazole Compounds That Inhibit Anaplastic Lymphoma Kinase. WO 2012018668 A1, 2012, World Intellectual Property Organization.

[18] Thottumkara, K. V.; Thottumkara, A. P. User- and Eco-Friendly Hypervalent Iodine Reagent and Method of Synthesis. US 20040030187 A1, 2004, United States Patent and Trademark Office.

[19] Barda, D. A.; Mader, M. M. PI3 Kinase/mTOR Dual Inhibitor. US 20120184577 A1, 2012, United States Patent and Trademark Office.

[20] Ashwell, S.; Gero, T.; Ioannidis, S.; Janetka, J.; Lyne, P.; Oza, V.; Springer, S.; Su, M.; Yu, D. Substituted Thiophenes and Uses Thereof. WO 2005016909 A1, 2005, World Intellectual Property Organization.

[21] Lu, Z.; Yoon, T. P. Visible Light Photocatalysis of [2+2] Styrene Cycloadditions via Energy Transfer. Angew. Chem. Int. Ed. 2012, 51, 10329.

[22] Collier, P. N.; Campbell, A. D.; Patel, I.; Raynham, T. M.; Taylor, R. J. Enantiomerically pure alpha-amino acid synthesis via hydroboration-suzuki cross-coupling. J. Org. Chem. 2002, 67, 1802-1815.

[23] Amin, R.; Chen, J. X.; Cotterill, I. C.; Emrich, D.; Ganley, D.; Khmelnitsky, Y. L.; Yang, Q. Improved Synthesis of the C16–C20 Segment of Resolvin E1 Using Enantioselective Ketone Reduction and Lipase-Catalyzed Resolution. Org. Process Res. Dev. 2013, 17, 915-920.

[24] Goto, T.; Urabe, D.; Isobe, Y.; Arita, M.; Inoue, M. Total synthesis of four stereoisomers of (5Z, 8Z, 10E, 14Z)-12-hydroxy-17,18-epoxy-5,8,10,14-eicosatetraenoic acid and their anti-inflammatory activities. Tetrahedron 2015, 71, 8320-8332.

[25] Pflästerer, D.; Rettenmeier, E.; Schneider, S.; de Las Heras Ruiz, E.; Rudolph, M.; Hashmi, A. S. K. Highly Efficient Gold-Catalyzed Synthesis of Dibenzocycloheptatrienes. Chem. Eur. J. 2014, 20, 6752-6755.

[26] Austad, B. C.; Calkins, T. L.; Chase, C. E.; Fang, F. G.; Horstmann, T. E.; Hu, Y.; Watanabe, T. Commercial manufacture of Halaven®: chemoselective transformations en route to structurally complex macrocyclic ketones. Synlett 2013, 24, 333-337.

[27] Bravo, P.; Fustero, S.; Guidetti, M.; Volonterio, A.; Zanda, M. Stereoselective Mannich-type reaction of an acyclic ketimine with a substituted chlorotitanium enolate: efficient approach to d-erythro-α-trifluoromethyl-β-hydroxyaspartic units. J. Org. Chem. 1999, 64, 8731-8735.

[28] Tsuritani, T.; Mizuno, H.; Nonoyama, N.; Kii, S.; Akao, A.; Sato, K.; Mase, T. Efficient synthesis of 1,4-diaryl-5-methyl-1,2,3-triazole, a potential mGluR1 antagonist, and the risk assessment study of arylazides. Org. Process Res. Dev. 2009, 13, 1407-1412.

[29] Harter, M.; Kosemund, D.; Delbeck, M.; Kalthof, B.; Wasnaire, P.; Submeier, F.; Lustig, K. Heterocyclylmethyl-Thienouracile as Antagonists of the Adenosine-A2B-Receptor. US 20180065981 A1, 2018, United States Patent and Trademark Office.
